# Stimuli‐Responsive Aggregation‐Induced Emission Materials for Tumor Theranostics: Design Strategies and Biomedical Applications

**DOI:** 10.1002/smsc.70339

**Published:** 2026-07-13

**Authors:** Zhaohua Xu, Deyue Kong, Xiaoru Li, Miao Zhu, Haili Yan, Chenyang Zhang, Xiaochun Wang, Duiping Feng, Jiangfeng Du

**Affiliations:** ^1^ Department of Medical Imaging Shanxi Key Laboratory of Intelligent Imaging First Hospital of Shanxi Medical University Taiyuan Shanxi Province China; ^2^ School of Traditional Chinese Medicine Shanxi University of Chinese Medicine Jinzhong Shanxi Province China; ^3^ College of Clinical Medicine Inner Mongolia Medical University Hohhot Inner Mongolia China; ^4^ School of Pharmacy Shanxi Medical University Taiyuan Shanxi Province China; ^5^ Department of Radiation Oncology Peking University Third Hospital Beijing China; ^6^ Key Laboratory of Cellular Physiology at Shanxi Medical University Ministry of Education Taiyuan Shanxi Province China; ^7^ Department of Oncological and Vascular Intervention First Hospital of Shanxi Medical University Taiyuan Shanxi Province China

**Keywords:** aggregation‐induced emission (AIE), bioimaging, nanomedicine, stimuli‐responsive materials, tumor theranostics

## Abstract

Smart‐responsive aggregation‐induced emission (AIE) materials have emerged as promising bio‐nanomaterials for tumor diagnosis and therapy due to their enhanced fluorescence in aggregated states, overcoming the aggregation‐caused quenching of traditional fluorophores. These materials enable the construction of efficient imaging and therapeutic platforms in complex biological environments. Stimuli‐responsive AIE systems can be categorized into pH‐, redox‐, thermo‐, enzyme‐responsive, and externally triggered types. Despite significant progress in AIE‐based tumor imaging and therapy, critical challenges in their rational design and biomedical application remain insufficiently addressed. This review summarizes recent advances in stimuli‐responsive AIE materials for tumor theranostics, focusing on their design strategies, functional mechanisms, and representative applications. Current limitations and future perspectives are also discussed to guide the development of next‐generation AIE‐based platforms.

## Graphical abstract

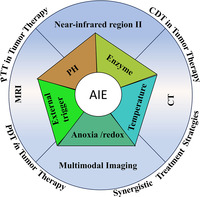

## 1 Introduction

Cancer has become one of the leading causes of death globally, with 18.1 million new cases and 9.6 million deaths worldwide in 2018, projected to reach 29.4 million new cases and 16.7 million deaths by 2024 [1]. Despite significant medical advances, traditional therapies including surgery, chemotherapy, and radiotherapy often cause damage to normal tissues due to insufficient targeting, severely affecting patients’ quality of life. In addition to traditional therapies, immunotherapy has demonstrated clinical efficacy in select cancer subtypes, but it faces challenges such as high costs, low response rates, and immune‐related adverse effects [2]. Besides, tumor heterogeneity, complex microenvironments, and treatment resistance result in a disappointing long‐term therapeutic efficacy for the above‐mentioned therapeutic method. So new breakthroughs in the efficient, safe, and highly specific therapeutic approaches deserve continuous attention. In the last few years, the “theranostic” concept has been widely proposed and extensively studied, providing an important direction for precise cancer diagnosis and treatment by combining precise tumor imaging with targeted therapy, which can achieve comprehensive coverage from early diagnosis to dynamic treatment monitoring [3]. This approach enables real‐time assessment of treatment responses and precision targeted therapy, significantly improving imaging performance/therapeutic efficiency and minimizing the adverse effects. It is believed that theranostic platforms hold promise for achieving novel, efficient, and precise tumor imaging/treatments.

In common theranostic platforms, stimuli‐responsive aggregation‐induced emission (AIE) materials show tremendous application potential in cancer theranostics due to their unique optical properties and design flexibility. Compared with traditional fluorescent materials that often suffer from aggregation‐caused quenching (ACQ), AIE materials emit minimal fluorescence in dilute solutions but exhibit significantly enhanced fluorescence in aggregated states [4, 5], which is mainly attributed to their special structure–activity relationships [6]. In dilute solutions, molecular motions lead to nonradiative energy dissipation; in aggregated states, restricted molecular motions cause excited‐state energy to be released as fluorescence [7].

This characteristic enables AIE materials to maintain high stability and signal‐to‐noise ratio in complex biological environments, making them particularly suitable for bioimaging and theranostics [8]. Despite their unique optical advantages in bioimaging and theranostics, traditional AIE materials often face significant challenges in effectively targeting tumor tissues, severely affecting the luminescence efficiency and theranostic efficacy. Active targeting functionalization technology by covalent conjugation of targeting moieties is often employed in the design of tumor‐targeting strategies; however, covalent conjugation of targeting moieties usually requires complex synthesis and may disrupt the aggregation behavior of AIE materials, leading to reduced fluorescence quantum yield. To overcome this challenge, in recent years, various strategies have been proposed to obtain tumor‐specific AIE materials. Of these, one of the promising approaches is to construct stimulus‐responsive AIE materials. Here, the AIE materials can be designed as responsive platforms, mainly including endogenous and exogenous stimuli‐responsive platforms (acidic pH, hypoxia, enzymatic activity, and redox signals) and exogenous stimuli‐responsive platforms (light, magnetic fields, and ultrasound) [5]. Stimulus‐responsive AIE materials can achieve better light‐emitting performance and realize more prominent precise imaging and targeted therapy.

**SCHEME 1:**
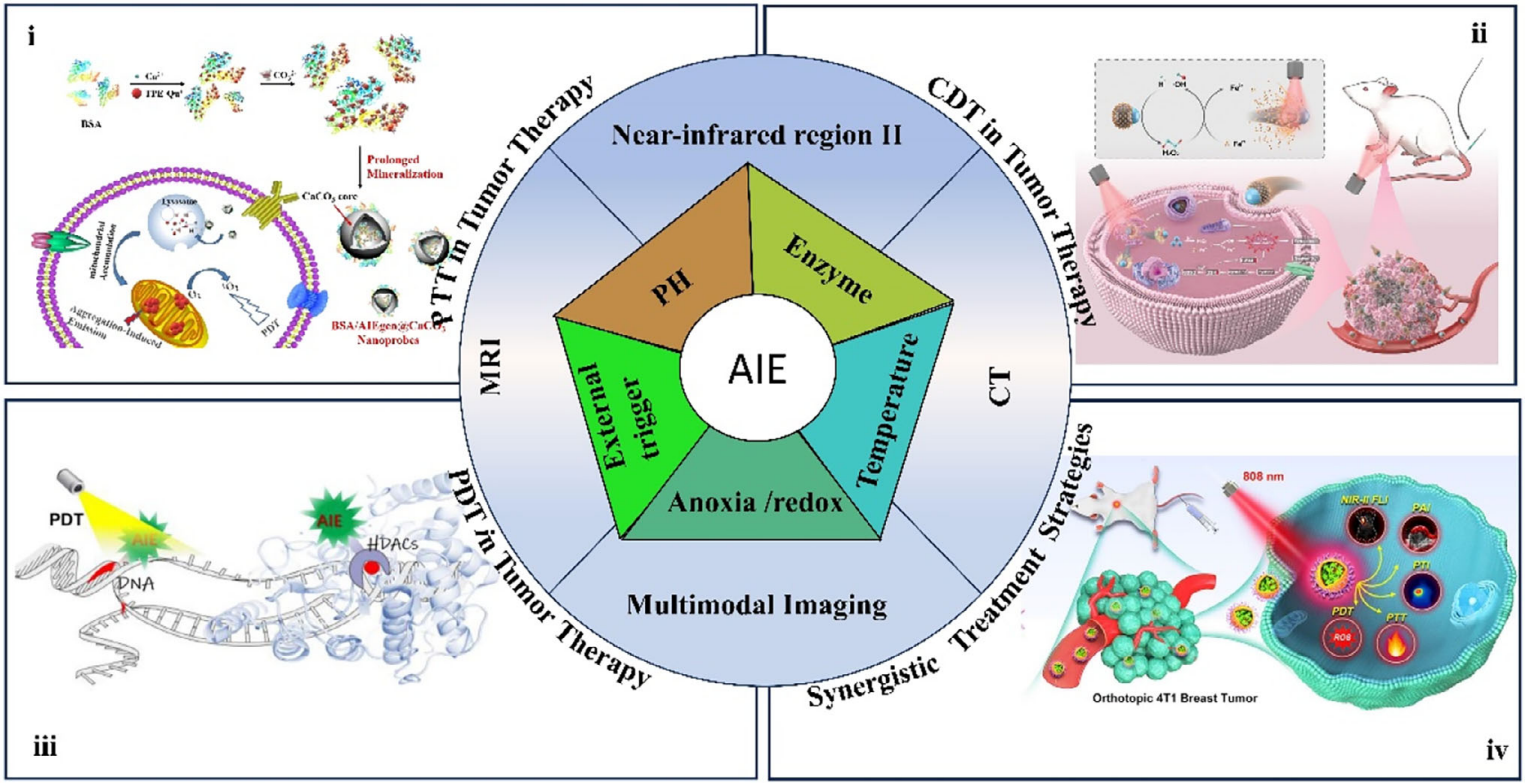
pH, enzyme, hypoxia/redox, temperature, and external trigger‐responsive AIE materials for tumor imaging and therapy. (i) Reproduced with permission [13]. Copyright 2021, American Chemical Society. (ii) Reproduced with permission [52]. Copyright 2024, Wiley‐VCH GmbH. (iii) Reproduced with permission [96]. Copyright 2022, Wiley‐VCH GmbH. (iv) Reproduced with permission [25]. Copyright 2023, American Chemical Society.

Currently, several reviews have summarized the progress of AIE materials and cancer theranostics from different perspectives. These previous reviews have provided valuable insights into the fundamental principles, material systems [9], and biomedical applications of AIE‐based platforms [10], including discussions related to stimulus‐responsive behaviors [11]. Nevertheless, a systematic and specialized review focusing on stimulus‐responsive AIE systems for tumor theranostics remains highly desirable. To lay a solid theoretical foundation for subsequent discussion on various responsive AIE platforms, we first detail the inherent structure–property relationship of AIEgens before classifying different stimulus‐responsive types. Against this background, this review presents a comprehensive and up‐to‐date overview of stimulus‐responsive AIE materials for tumor imaging and therapy. We provide a well‐organized classification framework for endogenous, exogenous, and multistimulus‐responsive systems, carry out reasonable comparative analysis of different response strategies, and further discuss the design principles, working mechanisms, structure–performance relationships, and clinical translation potential. This review aims to offer a clear, standardized, and in‐depth perspective for the rational design and future development of stimulus‐responsive AIE theranostic platforms (Scheme 1).

## 2 Structure–Property Rules of Stimulus‐Responsive AIE Molecules

Prior sections have summarized the research progress of stimulus‐responsive AIE materials and their translational prospects in tumor theranostics. To systematically elaborate the intrinsic correlation between molecular architecture and photophysical/biological performances, this chapter dissects how core conjugated skeletons, spatial conformation, side‐chain engineering, electrostatic modification, and targeted ligand tuning manipulate fluorescence emission, HOMO–LUMO energy levels, ROS production, aqueous aggregation behavior and in vivo tumor enrichment efficiency from multiple dimensions.

### 2.1 Regulation of Physicochemical Properties via Conjugated Backbone and D–π–A Push–Pull Electronic Architecture

The extension of conjugated length, selection of heteroaromatic rings, and rational construction of donor–*π*–acceptor (D–*π*–A) frameworks dominate frontier orbital distribution, optical emission, and ROS‐ generating capacity of AIE luminogens (AIEgens). Incorporating aromatic rings (benzene, thiophene) along the molecular backbone expands *π*‐electron delocalization and narrows the HOMO–LUMO bandgap, consecutively red‐shifting absorption and emission from visible light toward NIR‐I and even NIR‐II windows to improve deep‐tissue penetration for in vivo bioimaging [12].

Within D–*π*–A systems, the electron‐donating (D, e.g., triphenylamine (TPA)) and electron‐withdrawing (A, e.g., pyridinium, cyano) moieties govern intramolecular charge transfer (ICT) strength directly. Weak D/A combinations favor radiative fluorescence decay upon photoexcitation, yielding high aggregated fluorescence quantum yields superior for high‐contrast tumor fluorescence imaging [13]. Strengthened D–A interaction accelerates intramolecular charge separation and reduces singlet–triplet energy splitting (Δ*E*_ST_). When Δ*E*_ST_ falls within the optimal range of 0.3–0.8 eV, accelerated intersystem crossing (ISC) converts abundant singlet excitons to triplet states, sacrificing fluorescence quantum yield yet drastically elevating ROS productivity [13].

In terms of ROS subtypes, donor‐rich D–A configurations prefer electron‐transfer‐mediated Type I radicals (·OH, O_2_^−^·) independent of ambient oxygen, making them well‐suited for hypoxic solid tumors; acceptor‐dominant skeletons rely on energy‐transfer pathway to produce oxygen‐dependent Type II singlet oxygen (^1^O_2_), which is more applicable for well‐oxygenated superficial malignancies [14].

### 2.2 Modulation of Molecular Aggregation via Core Spatial Conformation and Side‐Chain Functionalization

Classic propeller‐shaped tetraphenylethylene (TPE) core possesses large dihedral angles to suppress tight intermolecular *π*–*π* stacking, fundamentally overcoming notorious ACQ of conventional organic fluorophores and guaranteeing intense aggregated‐state luminescence; in contrast, planar fused aromatics readily stack compactly to induce severe fluorescence attenuation [15].

Alkyl, carboxyl, and PEGylated side chains tune HLB to customize aggregate dimension: medium/long alkyl substituents introduce steric hindrance to restrain overpacking; hydrophilic carboxylate and PEG improve aqueous solubility to avoid uncontrolled oversized aggregation [15]. Tailored amphiphilic side chains confine nanoparticle diameter within 20–100 nm, matching the optimal size window for tumor enhanced permeability and retention (EPR) effect. Excess hydrophobic alkyl leads to aggregates over 100 nm that are rapidly sequestered and cleared by hepatic/spleen reticuloendothelial system; overhydrophilic modification yields particles smaller than 20 nm prone to fast renal excretion, both scenarios diminishing intratumoral accumulation in vivo [16].

### 2.3 Optimization of In Vivo Tumor Accumulation via Electrostatic and Targeted Ligand Decoration

Because of backbone and side‐chain design, ionic functionalization and bioligand conjugation further boost tumor enrichment from electrostatic interaction and specific receptor‐mediated active targeting perspectives. Cationic pyridinium/quaternary ammonium modification endows moderate positive surface potential to strengthen electrostatic adhesion and endocytosis by tumor cell membranes, yet excessive positive charge disrupts erythrocyte membrane integrity and triggers severe hemolysis; anionic sulfonate modification confers superior colloidal stability but increases nonspecific hepatic macrophage uptake to reduce intratumoral retention [17].

Covalent conjugation of RGD, biotin, or TPP ligands enables active tumor homing via overexpressed surface receptors on malignant cells, breaking the limitation of passive EPR‐dependent accumulation and elevating tumor enrichment ratio by 3–10 folds [18]. Additionally, heteroatom/heavy‐atom (N, S, halogen) doping accelerates ISC via spin–orbit coupling to enhance intracellular ROS production, whereas redundant heavy‐atom substitution causes persistent in vivo bioaccumulation and long‐term systemic toxicity [19].

### 2.4 Synergistic Effect of Multivariate Structural Parameters Determining Final Antitumor Efficacy

The backbone topology, spatial conformation, side‐chain composition, electrostatic status, and targeting modification function synergistically rather than independently, jointly defining the integrated in vitro imaging performance and in vivo antitumor potency of AIE nanoagents [20]. NIR‐shifted emission guarantees deep‐tissue optical penetration to circumvent insufficient therapeutic depth induced by strong visible‐light bio absorption; D–A‐regulated dual‐type ROS generation adapts variable intratumoral oxygen concentration to conquer hypoxia‐caused photodynamic therapy resistance; size‐optimized amphiphilic nanoparticles passively accumulate at tumor sites via EPR effect and reduce off‐target deposition in healthy visceral organs; targeted ligand engineering further amplifies tumor‐specific enrichment, lowering systemic adverse effects while elevating local intratumoral drug concentration [21].

Table 1 systematically summarizes property evolution, inherent drawbacks, and corresponding experimental data of this work alongside cited literature for all listed molecular modification tactics.

**TABLE 1:** Summary of structure–activity relationships of typical AIEgens.

Molecular structure	AIE skeletons	Structure‐dependent performance regulation	Main drawbacks	References
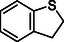	Conjugated *π*‐backbone (benzene/thiophene/fused heteroarene)	Longer conjugation/heterocycle → red shift ↑; Planar: *π*–*π* stacking, QY ↓; nonplanar: anti‐ACQ, QY ↑; Extended *π* & heteroatom → ISC & ROS ↑; NIR emission enhances deep‐tissue penetration for tumor imaging and therapy	Planar ACQ; overlong conjugation poorly soluble	[4, 5, 6, 22, 23]
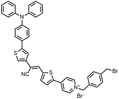	D–*π*–A push–pull electronic architecture (donor/acceptor/*π*‐bridge)	Stronger D–A → ICT ↑, red‐shift ↑ (NIR‐II available); Weak D–A: high QY; strong D–A: low QY, ISC & ROS ↑; D‐rich → Type I ROS; A‐rich → Type II ROS; NIR + dual ROS fit hypoxic/oxygen‐rich tumor	Excess D–A causes TICT quenching; weak D–A low absorbance	[11, 24, 25, 26]
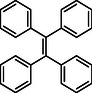	Sterically hindered twisted core (TPE/helical bulky aryl)	Large torsion → slight blue‐shift ↓; Steric hindrance inhibits stacking, QY ↑↑; Moderate twist → more ROS; over‐rigid → less ROS; Proper size matches EPR for high tumor enrichment	Over‐twisting reduces water solubility; too rigid weakens ROS	[27, 28, 29]
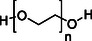	Aliphatic/polar side‐chain modification (long alkyl/hydroxyl/carboxyl/PEG)	Polar side chains barely shift wavelength; Proper alkyl → higher QY; hydrophilic PEG controls size 20–100 nm; Optimal amphiphile favors EPR enrichment	Long alkyl precipitates; excess hydrophilic hinders endocytosis	[10, 30, 31, 32]
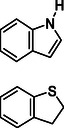	Targeting ligand conjugation (RGD/folate/TPP/galactose)	Ligand hardly alters spectrum/size; Active targeting × 3^−10^ tumor enrichment, indirect ROS elevation	High cost; ligand degraded in vivo	[30, 31]

## 3 Stimuli‐Responsive AIE Materials for Tumor Theranostics: Design Strategies and Biomedical Applications

### 3.1 pH‐Responsive AIE Materials in Tumor Applications

#### 3.1.1 Design Strategies for pH‐Responsive AIE Materials

The acidic characteristics of the tumor microenvironment (TME) arise from the enhanced glycolysis and lactic acid accumulation mediated by Warburg effect. This unique microenvironment fosters tumor progression through multiple mechanisms: first, it activates matrix metalloproteinase (MMP)‐mediated degradation of extracellular matrix; second, it reduces the ionization of chemotherapeutic drug and inhibits DNA repair pathways [24]; simultaneously, it weakens immune responses by inhibiting T cell receptor signal transduction through H^+^‐dependent mechanisms [33]. Based on these pathological features, constructing pH‐responsive AIE materials can facilitate tumor‐specific theranostics, with design strategies primarily based on four mechanisms.

##### 3.1.1.1 Protonation/Deprotonation Mechanisms

Protonation/deprotonation equilibria constitute the fundamental regulatory mechanism governing the luminescent behavior of pH‐responsive AIE materials. When ionizable functional groups on molecules undergo protonation reactions, they influence molecular orbital energy levels, charge distribution, and intermolecular electrostatic interactions, thereby controlling the transition between molecularly dissolved states and aggregated phases, ultimately leading to enhanced or diminished fluorescence [34]. The pathophysiological pH discrepancy distinguishes extracellular TME (pH 6.2–6.9) from intracellular tumor cytoplasm (maintained at pH 7.2–7.4 via MCT/NHE1 pH‐homeostasis transporters) and lysosomal endosomal compartments (pH 4.5–5.5 after endocytosis), creating a tiered pH gradient to trigger selective activation of pH‐sensitive AIE probes. Carboxylate‐bearing moieties with pKa of 4.5–5.5 experience a rise in protonation fraction from ∼20% to ∼90% when ambient pH drops from 6.5 down to 5.0, which mainly occurs within acidified endolysosomal vesicles rather than bulk extracellular tumor matrix under typical solid‐tumor acidosis [35].

**FIGURE 1:**
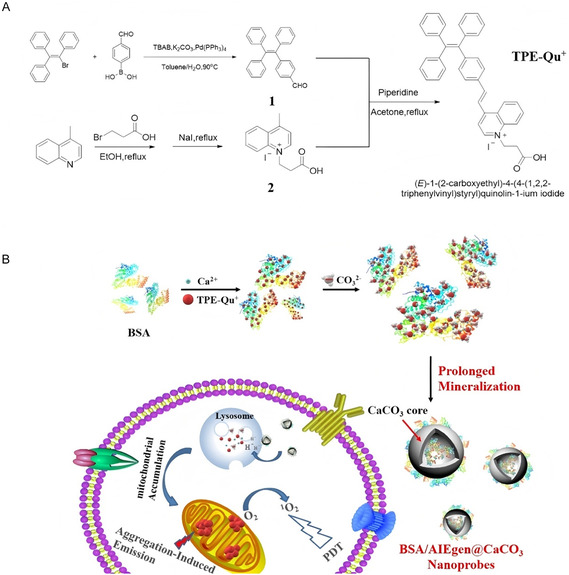
(A) Synthetic route of the TPE‐Qu^+^ molecule; (B) schematic Illustration of the preparation and working principle of the BSA/AIEgen@CaCO_3_ Nanoprobes. Reproduced with permission [27]. Copyright 2021, American Chemical Society.

Protonation‐induced AIE activation involves a cascade of structural reorganization processes that collectively optimize the photophysical properties of the fluorophore system. Protonation events reduce molecular polarity and hydrophilicity, leading to decreased solvation energy and promoting desolvation‐driven aggregation processes. Simultaneously, charge neutralization after protonation eliminates intermolecular electrostatic repulsion, reduces molecular hydration shell thickness and solvation free energy, and subsequently drives hydrophobic‐driven molecular stacking to form thermodynamically favorable J‐type aggregates with an interplanar distance of 3.5–4.0 Å; RIM within ordered aggregates suppresses nonradiative vibrational relaxation to boost radiative fluorescence emission following core AIE mechanism [12]. These supramolecular assemblies exhibit restricted intramolecular motion and optimized electronic coupling, resulting in prolonged excited‐state lifetimes and suppression of nonradiative decay pathways, ultimately manifesting as enhanced quantum yield and emission intensity. The CaCO_3_@BSA‐TPE‐Qu^+^ nanosystem exhibits dual‐responsive behavior, integrating acid‐catalyzed inorganic dissolution kinetics with organic fluorophore activation, resulting in a quantum yield enhancement from 0.12 to 0.68. This achieves a remarkable signal‐to‐background ratio of 9.7:1 and shows promising potential as a lab‐scale theranostic prototype validated in relevant animal studies (Figure 1) [27].

##### 3.1.1.2 Amphiphilic Balance Transformation

Based on protonation/deprotonation equilibrium mechanisms, amphiphilic balance transformation serves as a core design strategy for AIE luminescent materials by regulating hydrophilic–hydrophobic ratios. At physiological pH 7.4, abundant ionized carboxylate/amino groups raise overall molecular hydrophilicity to keep AIE luminogens molecularly dispersed, where free intramolecular twisting consumes excited‐state energy via nonradiative decay and quenches fluorescence. Upon acid‐induced protonation of ionizable residues, reduced molecular hydrophilicity shifts amphipathic balance toward lipophilic dominance, triggering hydrophobic aggregation and RIM‐mediated AIE turn‐on. This balance directly influences material affinity with biological membranes, affecting membrane fluidity and transport efficiency for nanodrug carrier design [32].

pH acts as a key external stimulus, controlling molecular properties through ionizable group dissociation. According to the Henderson–Hasselbalch equation, pH adjustments influence carboxylic acid (‐COOH/‐COO^−^) and amino group (‐NH_2_/‐NH_3_^+^) protonation, altering surface charge density. Acidic conditions promote hydrophobic aggregation, while alkaline environments keep dispersion. This pH‐responsive mechanism capitalizes on tumor tissue acidosis resulting from the Warburg effect, enabling selective AIE activation in pathological microenvironments [35].

**FIGURE 2:**
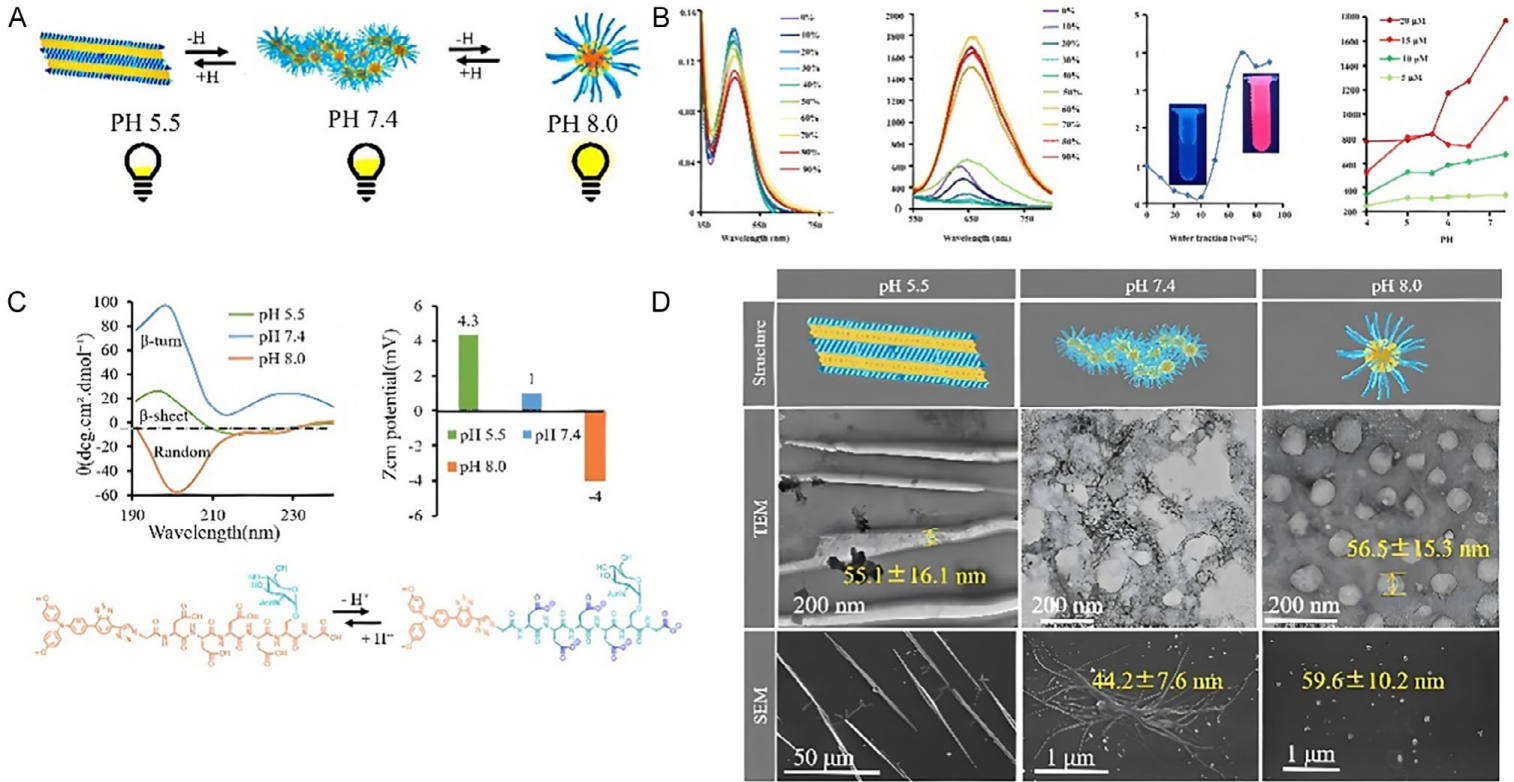
(A) Amphiphilic conversion of TGO. (B) The study of the photophysical properties of TGO includes the UV–vis absorption spectra and fluorescence spectra in different DMSO/H_2_O mixture solutions; the relative variation of fluorescence intensity *I*/*I*_0_ (*λ*_ex_: 470 nm, λem: 660 nm, concentration: 50 μM); and the fluorescence intensity (660 nm) of TGO at different concentrations and pH values. (C) The morphology and spectra of the nanoassemblies include circular dichroism spectra, spectra and zeta potential measurements at different pH values, and a schematic representation of the ionized state of TGO at pH 5.5 and pH 8.0. (D) TEM images and SEM images of the assembled morphology of TGO at different pH values. Reproduced with permission [32]. Copyright 2021, American Chemical Society.

The TGO probe system developed by Zhang et al. exemplifies the molecular engineering design of pH‐controlled amphiphilic balance transformation. This tetrahedral molecular configuration employs TPE as the hydrophobic core, with four aspartic acid tetrapeptide chains (Asp_4_ tetrapeptide) covalently linked to the periphery forming a hydrophilic corona. Quantitative evaluation reveals that under physiological pH 7.4 conditions, the material's critical aggregation concentration (CAC) is 45.2 μM with an HLB value of 14.2, presenting a hydrophilic‐dominated micellar dispersed state. At TME pH 5.0, Asp residue protonation reduces the HLB value to 8.7 and decreases CAC to 8.7 μM, triggering hydrophobic‐driven self‐assembly processes. The TGO probe achieves pH‐dependent amphiphilic conversion via aspartic acid protonation to achieve preferential AIE activation at acidic tumor sites in preclinical animal models (Figure 2) [32].

Based on this design principle, Deng's group achieved efficient enrichment of the AIE/Biotin‐M nanosystem in tumor tissues (15.8% ID/g) through integration of biotin‐streptavidin targeting modules, demonstrating a singlet oxygen quantum yield (^1^O_2_ quantum yield) of 0.72 in sonodynamic therapy (SDT). This design constructs an integrated theranostic platform encompassing diagnostic imaging, targeted delivery, and therapeutic response evaluation [22].

##### 3.1.1.3 Host–Guest Interaction Mechanisms

**FIGURE 3:**
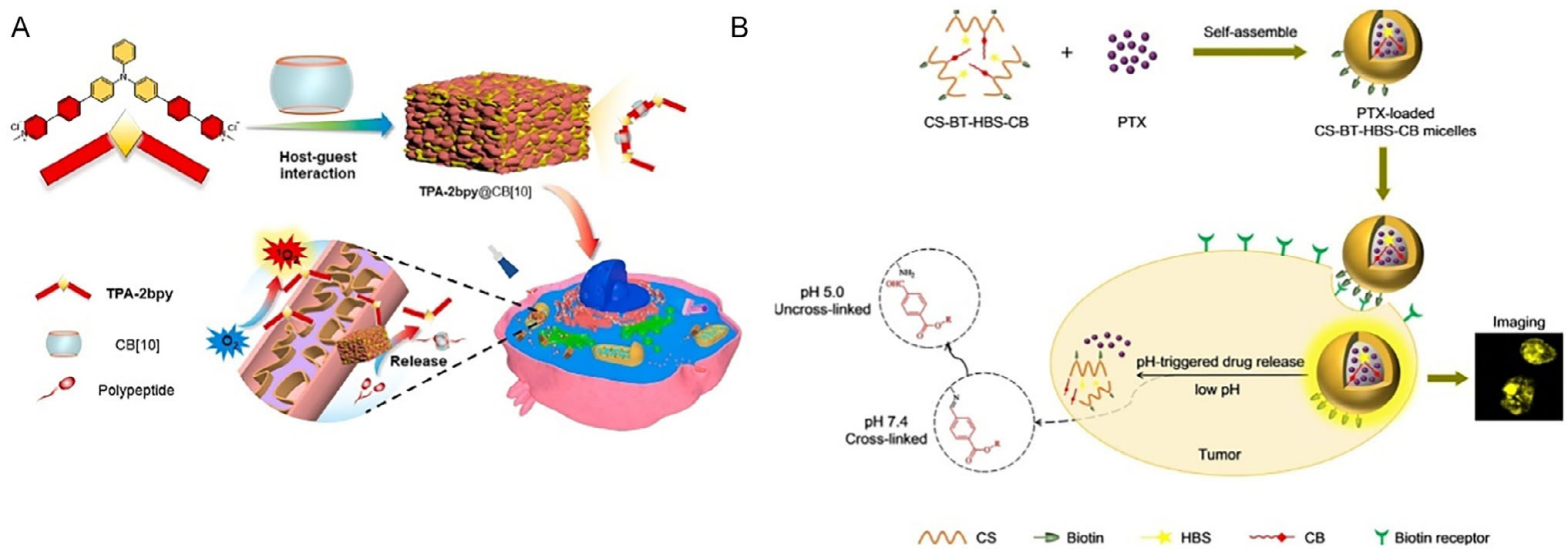
(A) The host–guest complexation between multicharged TPA and CB [5] constructs a supramolecular assembly. TPA‐2bpy, with its D*–π*–A structure, exhibits NIR imaging capability and ROS generation effects. The highly photosensitive TPA‐2bpy embedded in its supramolecular assembly releases in situ by competitively binding with endogenous substances after being internalized by cancer cells, achieving ablation at lower light intensities and delivery concentrations. Reproduced with permission [23]. Copyright 2024, Elsevier B.V. (B) CS‐BT‐HBS‐CB was prepared by introducing hydrophobic segments, tumor‐targeting ligands, acid‐sensitive bonds, and AIE fluorophores onto a chitosan backbone. Due to its amphiphilicity, the polymer can self‐assemble into micelles and encapsulate paclitaxel forming PTX‐loaded CS‐BT‐HBS‐CB micelles. Reproduced with permission [28]. Copyright 2022, Elsevier Ltd.

The host–guest interaction mechanism achieves precise regulation of pH‐responsive AIE materials through supramolecular chemistry principles. The core of this strategy lies in utilizing specific recognition between macrocyclic compounds (such as cucurbiturils and cyclodextrins) and AIE molecules to form environment‐responsive supramolecular assemblies. Single‐crystal X‐ray diffraction confirms the internal cavity of cucurbituril hosts has an inner diameter of ∼7.3 Å, which sterically encapsulates dual 2,2′‐bipyridine fragments. Host–guest confinement restricts the free torsional rotation of TPA skeletons, decreasing the dihedral torsion angle from 35° to 12°. Such rigidified molecular conformation blocks nonradiative deactivation pathways and elevates fluorescence quantum yield by around eight‐fold via canonical RIM‐based AIE activation (Figure 3A) [23]. Chitosan‐based and cucurbituril‐confined supramolecular AIE systems both utilize host–guest binding to construct acid‐labile assemblies; acidic cleavage disassembles the complexes to release free AIE fluorophores for tumor visualization and synergistic therapy (Figure 3B) [28].

The CS‐BT‐HBS‐CB system developed by Xu et al. demonstrates another innovative design concept for precise pH response via dynamic covalent chemistry. First, the benzylimine bond remains stable at physiological pH (hydrolysis half‐life *t*_1__/__2_ = 24 h) but rapidly breaks in the lysosomal acidic environment (*t*_1__/__2_ = 2.5 h); second, biotin modification enhances the material's binding capacity with biotin receptor‐overexpressing tumor cells by 5.7‐fold. Fluorescence lifetime imaging shows that this system has a fluorescence lifetime of 4.8 ns in tumor sites, significantly higher than normal tissues (1.2 ns). More prominently, with the aid of exactly controlling chitosan's deacetylation degree (85%–95%), researchers can accurately control nanoparticle surface potential (+15 to +35 mV), thereby optimizing their interaction efficiency with cell membranes (Figure 3B) [29]. Recently, Liu et al. developed the supramolecular AIE photosensitizer system 2SC‐3/CB [8] by encapsulating donor*–π*–acceptor structured AIEgen SC‐3 with cucurbit [8]uril (CB [8]); this assembly enhances ^1^O_2_ generation via restricting SC‐3's intramolecular motion, depletes tumor‐overexpressed spermine through CB [8]‐mediated host–guest interaction, and amplifies the antitumor efficacy of hypoxia‐activated chemotherapeutic tirapazamine in MDA‐MB‐231 breast cancer cells, serving as a promising synergistic strategy for hypoxic solid tumors [36].

##### 3.1.1.4 Dynamic Aggregation Effects

**FIGURE 4:**
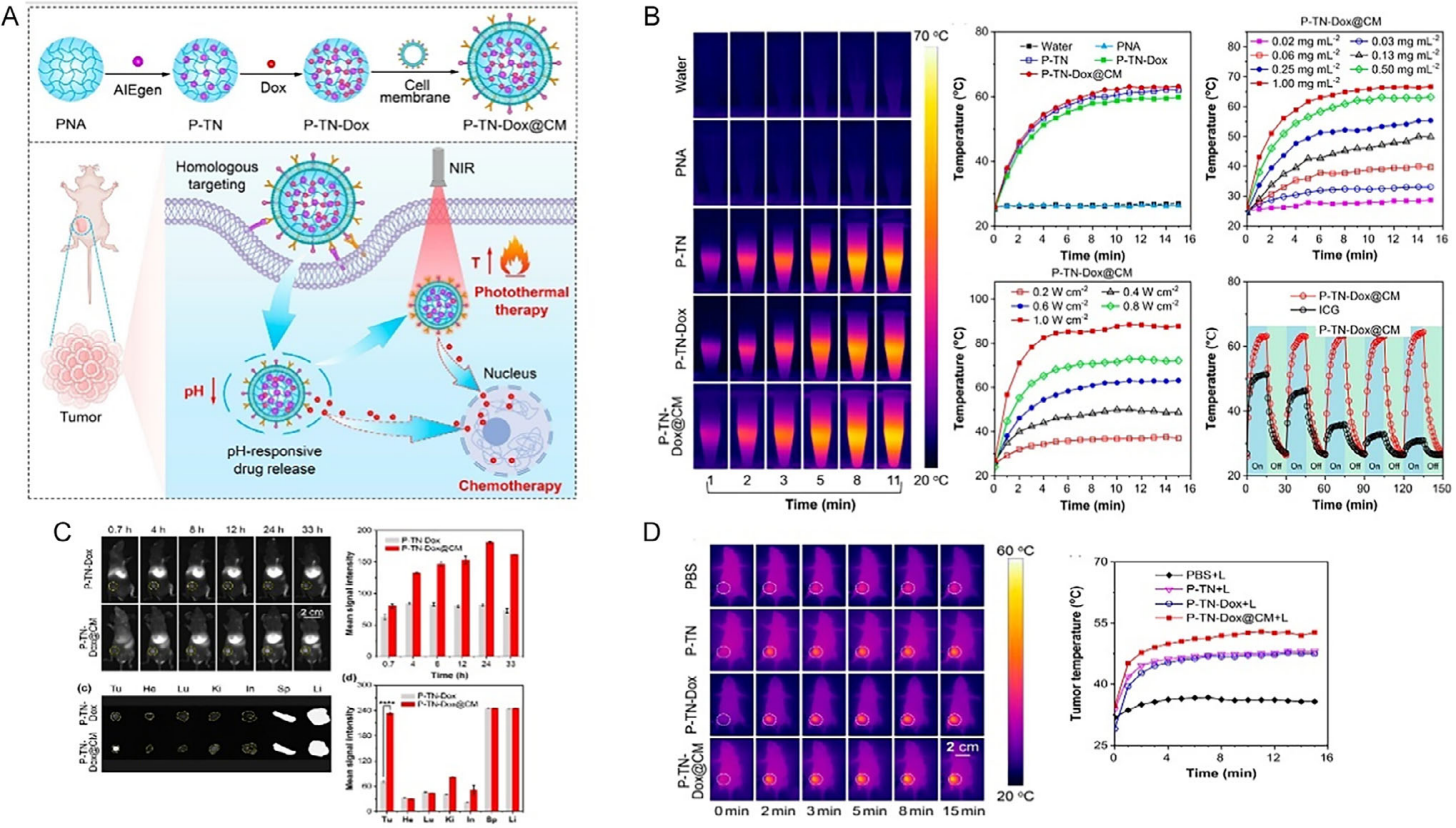
(A) AIE nanogels formulation process and schematic illustration of the homologous targeting and chemo‐photothermal synergistic therapy using the P‐TN‐Dox@CM nanogels. (B) Infrared thermal images and temperature change profiles of water, PNA, P‐TN, P‐TN‐Dox, and P‐TN‐Dox@CM nanogels in water (0.5 mg mL^−1^ based on PNA) under 808 nm laser irradiation (0.6 W cm^−2^) for 15 min. (C) In vivo and ex vivo fluorescence images and mean fluorescence signal intensity of tumors in 4T1 tumor‐bearing mice post intravenous injection of P‐TN‐Dox and P‐TN‐Dox@CM nanogels. (D) In vivo infrared thermal images and tumor region temperature curves of 4T1 tumor‐bearing mice irradiated by 808 nm laser for 15 min post intravenous injection of PBS, P‐TN, P‐TN‐Dox, and P‐TN‐Dox@CM nanogels, respectively. Reproduced with permission [25]. Copyright 2023, American Chemical Society.

Multiple inorganic and polymeric AIE nanosystems undergo pH‐driven reversible size and surface potential variation, enabling in situ fluorescence activation and controlled cargo release inside acidic TME [37]. Zhang's team designed P‐TN‐Dox@CM nanogels with temperature/pH dual‐responsive properties, which integrate AIE photothermal agents with cell membrane camouflage. Under acidic conditions, these nanogels swell (80 → 220 nm), release doxorubicin rapidly (80% within 5 min), and amplify fluorescence 7.2‐fold, boosting tumor regression rates from 30% to 92% in 4T1 models while enabling real‐time drug tracking (Figure 4) [25]. Du et al.'s NAB@DD‐DC liposomes address multiple challenges via charge reversal for prolonged circulation (9.7 h half‐life) and acidic‐tumor targeting (6.8‐fold uptake increase). This system triggers mitochondrial G‐quadruplex formation, enabling high‐contrast imaging (18:1 signal‐to‐noise ratio), ion interference therapy (15‐fold Na^+^ increase), and immunogenic cell death (ICD) (78% calreticulin exposure), providing one optional research idea [26].

#### 3.1.2 Applications in Tumor Imaging

Near‐infrared region II (NIR‐II, 1000–1700 nm) fluorescence imaging technology provides an important foundation for pH‐responsive AIE materials in tumor imaging due to excellent tissue penetration and high signal‐to‐noise ratio [31].

For dual‐modality imaging systems, Ding et al. designed PE‐Py‐PAI system combining photoacoustic imaging with fluorescence imaging, achieving 820 nm maximum absorption and 42% photothermal conversion efficiency through molecular structure optimization, demonstrating 10.2 mm imaging depth and 78 μm spatial resolution in melanoma models [34]. Zhang et al.'s TPE‐I probe enables fluorescence‐computed tomography (CT) dual‐modality imaging via iodine modification, reaching 89% sensitivity and 93% specificity in liver cancer detection [30].

#### 3.1.3 Applications in Tumor Therapy

##### 3.1.3.1 PDT Applications

pH‐responsive AIE materials demonstrate unique advantages in PDT by triggering molecular aggregation in the TME (pH 6.5–5.0), significantly enhancing (^1^O_2_ quantum yield (ΦΔ). The TPE‐Ph‐NH_2_ photosensitizer developed by Yang's team achieves a ΦΔ of 0.82 at pH 5.0, 5.5 times higher than in neutral environments, realizing a 78% ± 6% tumor inhibition rate in 4T1 breast cancer models, exhibits better efficacy than partial reference photosensitizer within identical laboratory test conditions. The TPE‐Ros probe (pKa = 6.3) designed by Liu et al. achieves 6.8 times higher ^1^O_2_ production at tumors than normal tissues via pH/esterase dual‐response mechanisms, greatly improving the therapeutic window and reducing off‐target [7].

Recent advances focus on near‐infrared region II responsive AIE photosensitizers, such as TPE‐BODIPY (*λ*_em_ = 1050 nm), which can be capable of penetrating tissues 12 mm deep while enabling simultaneous therapy and monitoring [38]. The main challenge currently facing this field is limited light penetration depth in tissues. Solutions include combining conversion nanoparticles to convert deep tissue‐penetrable near‐infrared light into visible light that activates photosensitizers and developing two‐photon excitation AIE materials that can generate one high‐energy excited state using two low‐energy photons [39].

##### 3.1.3.2 PTT Applications

pH‐responsive AIE photothermal agents significantly enhance photothermal conversion efficiency (*η*) via protonation‐induced molecular planarization effects. The TPE‐TTQ material reported by Zhang et al. demonstrates a substantial increase in *η* from 32% to 58% at pH 5.0, rapidly elevating tumor local temperature to 56 °C under 1064 nm laser irradiation, achieving efficient thermal ablation. More advanced intelligent temperature‐controlled materials, such as TPE‐PNIPAM, automatically terminate the heating process at 42 °C through phase transition mechanisms, effectively avoiding thermal damage to surrounding normal tissues [40].

Recent trends combine PTT with immunotherapy: the AIE/Biotin‐M system developed by Deng's team generates ^1^O_2_ via ultrasound activation, inducing ICD and increasing CD8^+^ T cell infiltration in the TME by 3.2‐fold, activating systemic antitumor immunity [22].

##### 3.1.3.3 Drug Delivery Applications

pH‐responsive AIE carriers enable precise tumor delivery via charge reversal mechanisms. The NAB@DD‐DC liposome system designed by Du et al. rapidly transforms its zeta potential from −25 mV to +15 mV in the TME, enhancing tumor cell uptake efficiency six‐fold, significantly optimizing drug distribution in vivo [26]. For intracellular drug transport, organelle‐targeting systems (such as triphenylphosphonium (TPP)‐modified carriers targeting mitochondria) can enhance drug colocalization coefficients with target organelles to 0.87 [41], achieving subcellular‐level precise delivery and greatly improving drug utilization efficiency. Recent host–guest supramolecular systems (e.g., CB‐triphenylamine complexes) achieve >25 wt% drug loading and precise release triggered by endogenous polyamines. Similarly focusing on pH‐responsive AIE‐enabled drug delivery, Rupel et al. synthesized biocompatible cellulose‐TPE hydrazone self‐assembling nanomicelles (CE‐TPEHy‐NMs) loaded with doxorubicin (DOX); these AIE‐active nanomicelles (60 ± 17 nm unloaded, 86 ± 25 nm DOX‐loaded) showed high encapsulation efficiency (86%) and loading capacity (90%), pH‐dependent DOX release (11% at pH 7.4, 50% at pH 6.5, 80% at pH 4.5), and significant proliferation inhibition on MG‐63 human osteosarcoma cells at pH 6.5 (not pH 7.4), validating their potential as tumor‐targeted carriers [42].

#### 3.1.4 Key Challenges and Solution Strategies

**FIGURE 5:**
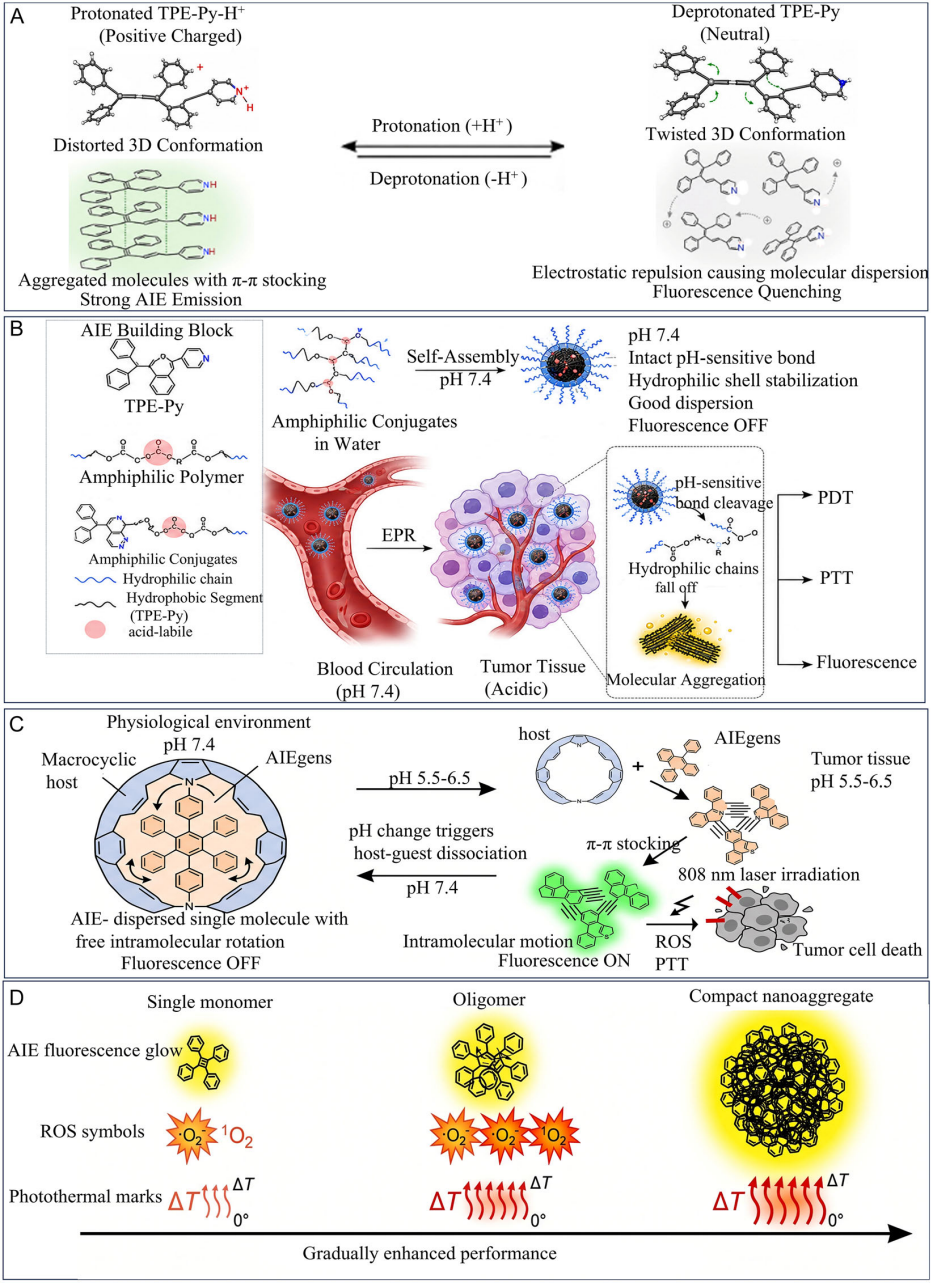
Schematic illustration of design principles and working mechanisms of representative pH‐responsive AIE materials for tumor theranostics. (A) Protonation/deprotonation regulation of TPE‐Py conformation and aggregation. (B) Amphiphilic pH‐responsive AIE nanoplatform for tumor theranostics. (C) pH‐responsive host–guest supramolecular AIE assembly. (D) Gradual enhancement of AIE performance with molecular aggregation.

Overall, pH‐responsive AIE materials for tumor theranostics fall into four major categories based on their working mechanisms: protonation–deprotonation regulation, amphiphilic balance transformation, host–guest supramolecular interaction, and pH‐triggered dynamic aggregation. Figure 5 intuitively illustrates the design principles, structural regulation modes, and performance advantages of typical pH‐responsive AIE systems, while Table 2 summarizes the response mechanisms, representative material systems as well as inherent pros and cons of different strategies. Although pH‐responsive AIE theranostic materials have achieved certain progress in molecular design and biomedical applications, multiple bottlenecks still restrict their clinical translation.

**TABLE 2:** Summary of four major pH‐responsive mechanisms of AIE materials for tumor theranostics.

Design principle	Response mechanism	Representative systems and references	References
Protonation/deprotonation	Utilize ionizable groups to regulate molecular charge, orbital and intermolecular interactions, switching molecular dispersion/aggregation states	CaCO_3_@BSA‐TPE‐Qu^+^ TPE‐Ph‐NH_2_	[7, 26]
Amphiphilic balance transformation	Modulate HLB via protonation to control molecular assembly	TGO AIE/Biotin‐M NAB@DD‐DC	[21, 25, 30]
Host–guest interaction	Construct supramolecular assemblies via specific recognition between macrocycles and AIE molecules	TPA‐CB [5] CS‐BT‐HBS‐CB 2SC‐3/CB [8]	[22, 28, 36]
Dynamic aggregation	Design inorganic/polymeric nanosystems with pH‐dependent size and surface potential changes	P‐TN‐Dox@CM CE‐TPEHy‐NMs NAB@DD‐DC	[24, 25, 41]

First, extracellular TME of most solid tumors is mildly acidified at pH 6.2–6.8 instead of strongly acidic conditions (pH 5.0–5.5) required for full protonation of most pH‐sensitive AIE moieties. This mismatch originates from inherent tumor intracellular pH homeostasis maintained via MCTs and Na^+^/H^+^ exchangers, which continuously pump metabolic lactate and excess H^+^ outward while preserving near‐neutral cytoplasmic pH; only endocytosed nanoparticles experience pH drop down to 4.5–5.5 within lysosomes after endosomal acidification [43]. This gap arises from tumor cells’ ability to maintain intracellular pH homeostasis through lactate transporters and pH regulatory systems. To address this, researchers have proposed metabolic regulation‐based acidification strategies, such as using carbonic anhydrase IX inhibitors to actively lower tumor pH or adopting gradient pKa designs that incorporate multiple acid‐sensitive functional groups to adapt to varying acidic microenvironments.

Second, the materials must remain stable under physiological conditions while achieving precise drug release in acidic tumor regions. Existing pH‐sensitive bonds often lack sufficient stability, leading to premature drug leakage. Solutions include developing intelligent locking mechanisms and dual‐response systems that combine pH‐sensitive units with enzyme‐cleavable sites to ensure controlled drug release under specific conditions. Biomimetic designs that simulate the pH changes during cellular endocytosis can also enable staged drug release, while incorporating pH memory functions helps prevent accidental release caused by transient pH fluctuations [44].

Furthermore, pH‐responsive AIE materials tend to aggregate and increase in particle size under acidic conditions, which, while enhancing tumor retention, limits their penetration in dense tumor tissues. Reversible size control strategies (e.g., “small entry–large retention” or “large entry–small dispersion”) show promise in improving tissue diffusion [45]. Finally, these materials must avoid nonspecific activation in nontumor acidic environments, such as gastric acid or inflammatory sites. Dual‐gated systems combining pH with enzyme or hypoxia responses can enhance tumor selectivity and minimize off‐target risks. In summary, through these strategies, relevant optimized designs may help improve partial existing drawbacks in subsequent laboratory exploration.

### 3.2 Hypoxia‐Responsive and Redox‐Responsive AIE Materials in Tumor Applications

Tumor tissues often exhibit a hypoxic microenvironment, with oxygen concentrations frequently below 5%, and in the core regions of tumors, the oxygen concentration can even drop to 0.1%. This hypoxic condition arises from the rapid proliferation of tumor cells and the imbalance of abnormal angiogenesis, causing metabolic waste accumulation and redox imbalance in the tumor core region [46]. This specificity is not only characterized by hypoxia but is also accompanied by significant fluctuations in the concentrations of reductive metabolites (such as glutathione (GSH)) and ROS, as well as the abnormal accumulation of reductive metal ions. GSH and thiols affect multiple tumor signaling pathways primarily by regulating the redox balance. Currently, common redox‐responsive mechanisms for the design of AIE materials targeting the TME, common redox response mechanisms include nitro‐reduction reactions, azo bond cleavage, ROS regulation, and metal ion reduction responses [47].

#### 3.2.1 Design Strategies for Hypoxia‐Responsive and Redox‐Responsive AIE Materials

##### 3.2.1.1 Nitro‐Reduction Response Mechanisms

Nitro groups are typical redox triggers in AIE materials. Nitroreductase (NTR) is selectively overexpressed in hypoxic tumor niches (pO_2_ < 10 mmHg) due to hypoxia‐dependent transcriptional regulation. Under low oxygen tension, prolyl hydroxylases lose catalytic activity and fail to initiate HIF‐1α ubiquitination and proteasomal degradation; stabilized HIF‐1α translocates into cell nucleus and binds to hypoxia response elements on NTR promoter to trigger NTR transcriptional upregulation [48]. Critically, NTR‐mediated nitro‐to‐amino reduction strictly requires intracellular nicotinamide adenine dinucleotide (NADH) as the reducing cofactor, while ambient molecular O_2_ competes for available NADH under normoxia to drastically suppress NTR catalytic efficiency, restricting effective nitro reduction exclusively within hypoxic tumor regions. NTR catalyzes nitro reduction to amino groups, generating important tumor metabolic substrates for nucleotide/protein synthesis, supporting tumor cell proliferation [49].

**FIGURE 6:**
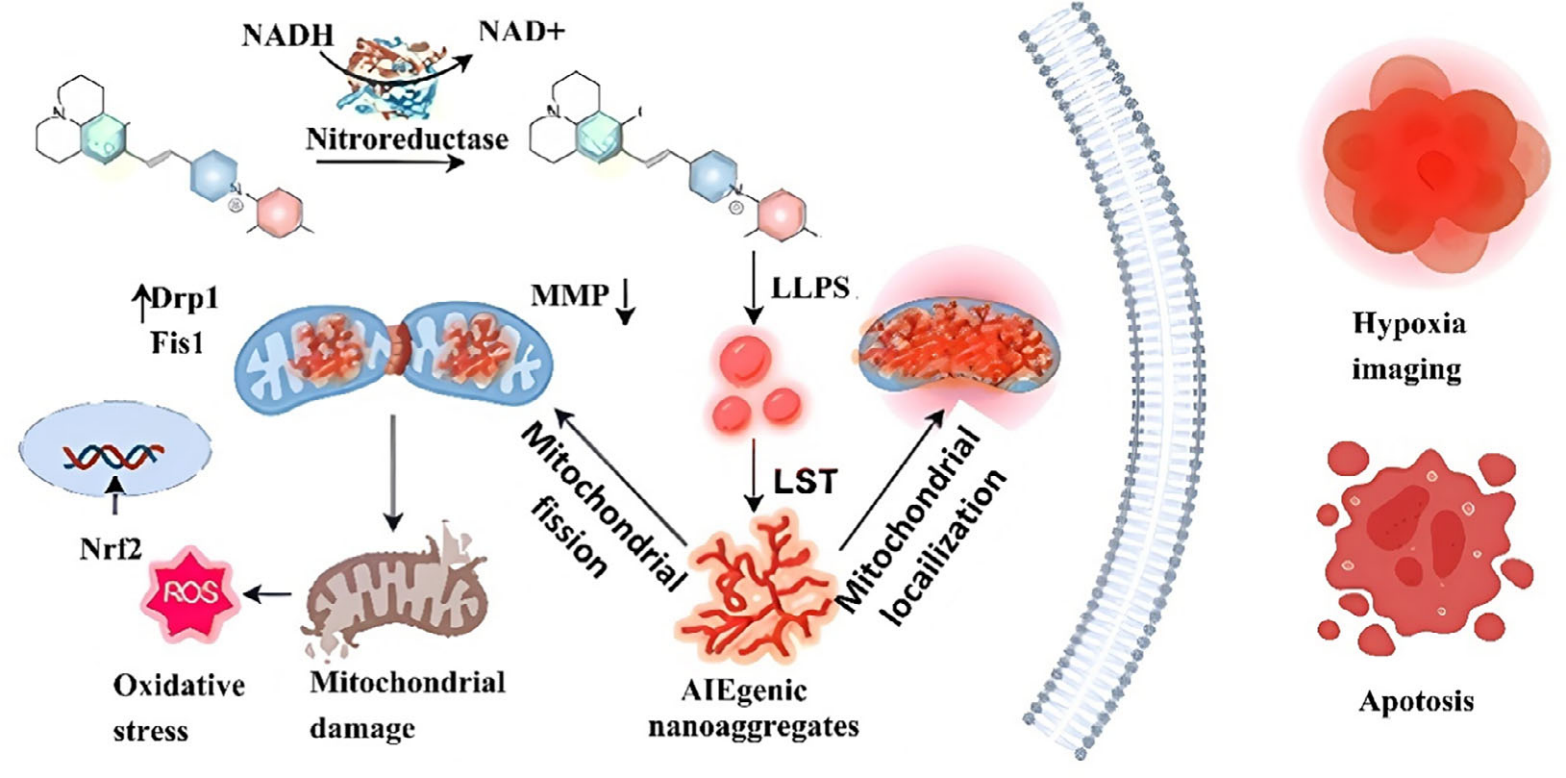
The small molecule DN6 undergoes nitroreductase‐responsive liquid–liquid phase separation, followed by liquid–solid transition, forming red‐emitting nanoaggregates that mimic toxic amyloid protein aggregation. These nanoaggregates target mitochondria, leading to mitochondrial fission and oxidative stress‐induced tumor cell apoptosis. At the same time, the reduction of DN6 (DN6R) allows imaging of tumor hypoxia through the AIE emission. Reproduced with permission [51]. Copyright 2025, Royal Society of Chemistry.

Based on nitro‐response design, AIE molecules typically introduce nitro groups as trigger points. Wang et al. developed an AIE photosensitizer based on TTVBA (2‐(4‐(Triisopropylsilyl)phenyl)‐5‐nitrobenzoic acid) and Biotin‐TTVBA (biotin‐modified TTVBA), which significantly enhanced its uptake ability in tumor cells through binding with biotin. The probe responds to reductive NTR in the TME, and its fluorescence characteristics are enhanced in the aggregated state, enabling tumor imaging and PDT [50]. Sain et al. developed pyridinium‐based compounds (DN1 to DN6), in which a dinitrobenzene segment was incorporated for fluorescence quenching and NTR recognition. After nitro reduction to amino groups, hydrogen bonding and *π* *–π* interactions were enhanced, promoting phase separation and liquid‐solid phase transition, resulting in AIE properties. Through this design, DN6R generated oxidative stress within tumor cells and induced apoptosis (Figure 6) [51].

##### 3.2.1.2 Glutathione‐Mediated Response Mechanisms

**FIGURE 7:**
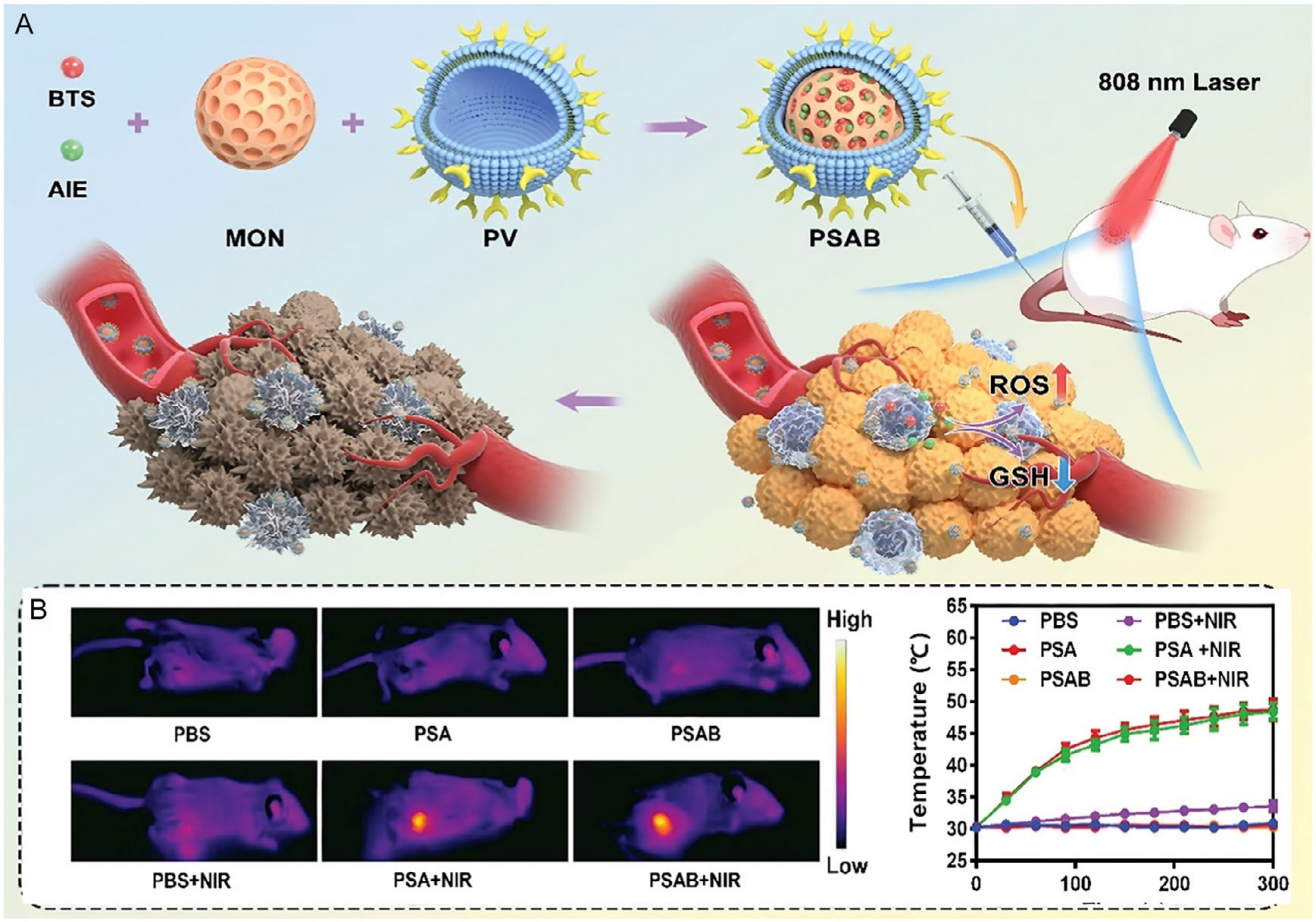
(A) Biomimetic nanosystem loading aggregation‐induced emission luminogens and SO_2_ prodrug for inhibiting tumor recurrence and metastasis after photothermal therapy. (B) Infrared images of the mice under laser irradiation for 5 min after the indicated treatments. Reproduced with permission [54]. Copyright 2024, Advanced Science published by Wiley‐VCH GmbH.

Metabolic reprogramming drastically elevates intracellular GSH concentration up to 5–15 mM inside malignant cells, whereas extracellular tumor matrix only contains less than 20 μM GSH; such a thousand‐fold concentration gradient facilitates preferential cleavage of disulfide‐containing AIE structures under in vitro and xenograft tumor experimental conditions. Abundant intracellular GSH functions as dominant endogenous antioxidant to quench photogenerated ROS, thereby compromising PDT therapeutic outcomes; rationally designed disulfide‐functionalized AIE photosensitizers consume surplus intracellular GSH upon bond cleavage to alleviate antioxidant‐mediated PDT resistance [52]. To address this, AIE photosensitizers are often functionalized with disulfide bonds, which undergo GSH‐triggered cleavage, depleting intracellular antioxidants while enabling ROS generation. Wang et al. developed an AIE photosensitizer (MBTB‐PA) that covalently binds GSH via sulfur‐alkyne click chemistry, reducing tumor antioxidant capacity and inducing ICD through ferroptosis and necroptosis [53]. Zhang et al. designed a biomimetic nanoplatform combining AIE, PTT, and SO_2_ gas therapy. The disulfide‐bonded mesoporous silica encapsulates AIE agents and SO_2_ prodrugs, enhancing PTT and gas therapy via GSH‐triggered bond cleavage while preventing tumor recurrence (Figure 7) [54].

##### 3.2.1.3 ROS Regulation Mechanisms

Photogenerated ROS are produced via two distinct photochemical pathways: Type‐II PDT relies on triplet–triplet energy transfer from excited photosensitizer to ground‐state ^3^O_2_ to yield singlet ^1^O_2_, which is strictly oxygen‐dependent and inefficient within hypoxic solid tumors; Type‐I PDT proceeds via electron transfer between excited fluorophores and surrounding biological substrates to generate O_2_•^−^ and •OH, featuring oxygen‐independent characteristics favorable for hypoxic tumor treatment. D–A molecular architecture accelerates ISC to increase triplet excited‐state population and improve overall ROS production yield for both pathways [55]. In cancer biology, ROS exhibit a concentration‐dependent duality: moderate levels promote tumor progression, while excessive accumulation triggers oxidative damage and cancer cell death. This therapeutic window has driven the development of advanced AIE photosensitizers with enhanced ROS‐regulating capabilities.

**FIGURE 8:**
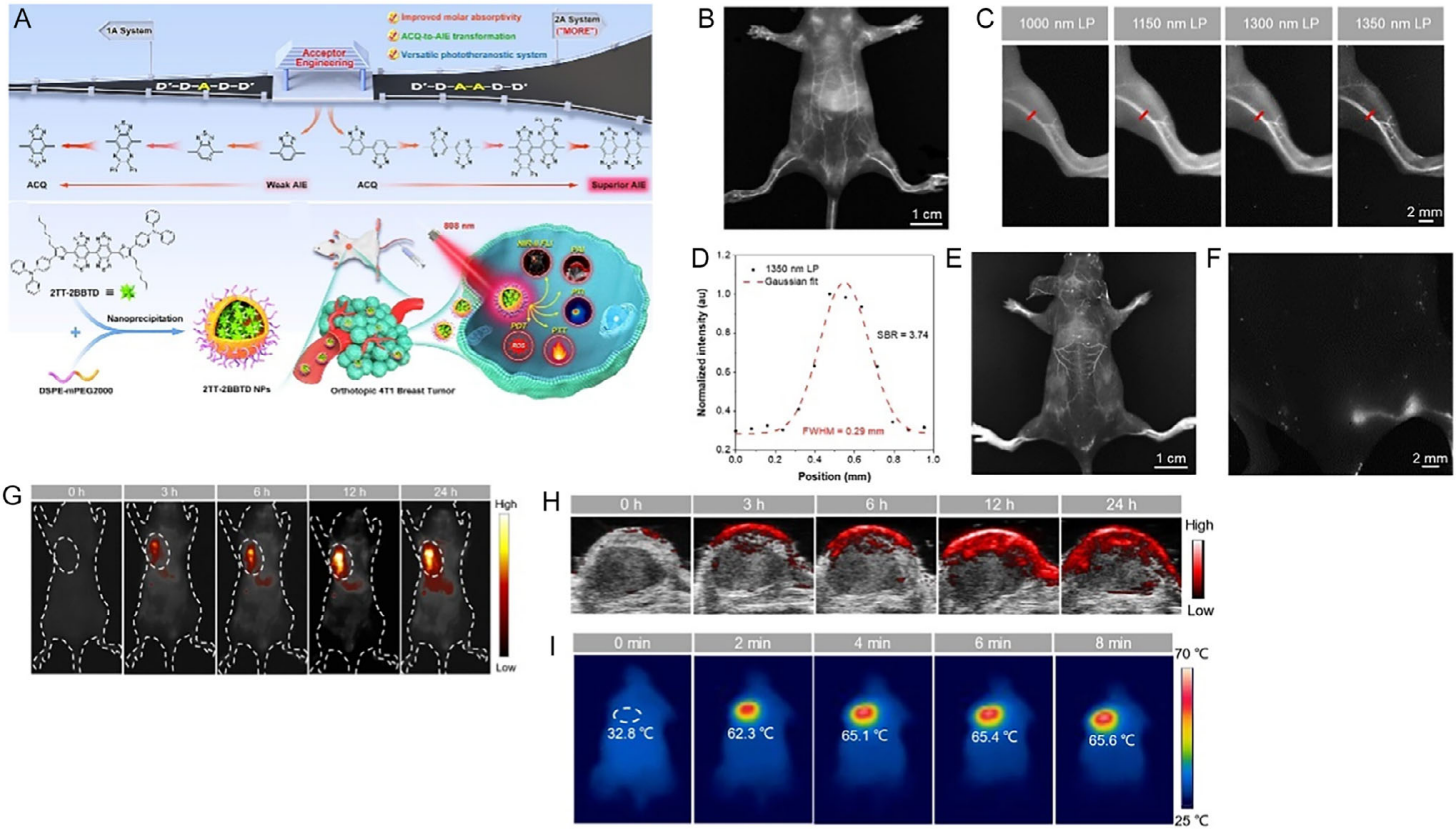
(A) Schematic of NIR‐II fluorescence/photoacoustic/photothermal tri‐modal imaging‐guided synergistic photodynamic/photothermal therapy using 2TT‐2BBTD NPs. (B–D) Whole‐body, hindlimb, and dorsal vascular NIR‐II fluorescence imaging of nude mice after 2TT‐2BBTD NP injection. (E) Fluorescence intensity distribution across blood vessels under 1350 nm long‐pass filtering. (F) Lymph node NIR‐II fluorescence imaging under 1150 nm long‐pass filtering. (G–H) Time‐dependent in situ NIR‐II fluorescence and photoacoustic imaging of 4T1 tumors after intravenous injection of 2TT‐2BBTD NPs. (I) In vivo photothermal imaging of 4T1 tumors under 808 nm laser irradiation at different time points postinjection. Reproduced with permission [56]. Copyright 2023, American Chemical Society.

Recent molecular engineering approaches have yielded significant improvements in ROS generation efficiency. The donor–acceptor system developed by Yang et al. demonstrates how dual‐receptor architectures showed better photophysical performance than partial single‐receptor analogues in cell and mouse assays (Figure 8) [56]. Building on these findings, researchers have further augmented ROS yields by incorporating heavy atoms like gold and iridium, which promote ISC through spin–orbit coupling effects. Notable examples include the TBP‐Au complex and DPTPzIr derivatives, which combine potent therapeutic action with NIR‐II imaging capabilities [57].

Targeted delivery strategies have emerged as a critical frontier in precision ROS modulation. Mitochondrial‐localizing agents such as Th‐M induce cancer cell death through organelle‐specific oxidative stress, while TME‐responsive systems like TPASIC‐PFH@PLGA NPs dynamically adapt to hypoxic conditions [41]. The development of bioorthogonal prodrug platforms represents another significant advancement, enabling simultaneous release of photosensitizers and chemotherapeutic agents for combined treatment modalities [58].

##### 3.2.1.4 Metal Ion Reduction Response Mechanisms

The abnormal elevation of reductive metal ions in the TME arises from disrupted tumor iron metabolism disorder and hypoxic conditions. On one hand, the rapid tumor cell proliferation demands large amounts of iron ions for DNA synthesis and cell cycle regulation, leading to overexpression of iron reductases. On the other hand, the hypoxic environment further promotes iron ion reduction by activating the Fenton reaction [59]. Intracellular overloaded Fe^2+^ initiates Fenton‐type catalytic reaction to decompose endogenous H_2_O_2_ into cytotoxic hydroxyl radicals and accelerate polyunsaturated fatty acid peroxidation. Moreover, accumulated Fe^2+^ indirectly suppresses GPX4 activity by depleting cellular GSH cofactor; inactivated GPX4 fails to eliminate accumulated lipid peroxides, which serves as the definitive biochemical hallmark driving the initiation and progression of ferroptotic tumor cell death.

Ferroptosis is often accompanied by mitochondrial dysfunction, ATP depletion, and autophagy inhibition. Additionally, Fe^2+^ inhibits the activity of GPX4, aggravating lipid peroxidation and promoting ferroptosis. Therefore, inducing ferroptosis is considered a potential antitumor therapeutic strategy [60]. Jiang et al. designed an iron ion‐responsive tetraphenylethene (TPE‐Fe) probe, which releases blue fluorescence signals through iron ion reduction reactions, can label hypoxic regions and realize combinatorial function in preclinical tumor models. Wang et al. developed a copper ion‐responsive probe that triggers fluorescence signal amplification via Cu^+^ redox reactions in liver cancer models, exhibiting excellent imaging performance.

#### 3.2.2 Applications in Tumor Imaging

Traditional imaging agents suffer significant signal attenuation in hypoxic tumor cores due to poor vascular perfusion and elevated reductive metabolites (e.g., GSH > 10 mM). Single‐modal redox‐responsive AIE materials alleviate part of the above drawbacks under animal experimental settings by utilizing tumor‐specific enzymatic triggers (e.g., NTR) or reductive microenvironments to activate fluorescence through controlled aggregation. For example, the TPE‐NO_2_ probe undergoes NTR‐catalyzed nitro‐to‐amine conversion in breast cancer cores, achieving a six‐fold fluorescence enhancement (quantum yield: 0.08 → 0.35) and tumor‐to‐normal tissue ratio of 7.2:1 [43]. Similarly, the TPE‐Az probe releases red‐emitting fluorophores (*λ*_em_ = 650 nm) in melanoma via GSH‐triggered azo bond cleavage, enabling precise delineation of necrotic regions. Recent advancements in single‐modal systems integrate NIR‐I/II optimization and “dual‐lock” designs, which require concurrent hypoxia (pO_2_ < 5 mmHg) and high GSH (>5 mM) for activation, reducing off‐target signals by 68% [61].

The inherent trade‐off between imaging depth and molecular resolution limits these conventional single‐modal approaches in characterizing heterogeneous hypoxic niches. Dual‐modal AIE probes overcome this limitation by coupling fluorescence specificity with deep‐penetrating modalities such as photoacoustic imaging and CT. TPE‐NO_2_/PAI hybrid nanoparticles leverage pH‐responsive aggregation to simultaneously provide 10.2 mm photoacoustic imaging depth (contrast ratio: 6.5:1) and 78 μm fluorescence resolution for mapping breast cancer microvasculature. Another breakthrough is an azo‐based NIR‐II probe (*λ*_em_ = 1050 nm), which synchronizes fluorescence (tumor SNR: 9.8) with CT contrast (ΔHU = 270) in gliomas to achieve submillimeter lesion detection (<0.5 mm error) [62].

Multimodal innovations extend these capabilities through time‐gated imaging strategies that temporally decouple fluorescence and photoacoustic signals, while zwitterionic surface modifications reduce hepatic accumulation to < 15% ID/g while maintaining tumor targeting efficiency (>5% ID/g). These integrated approaches enable comprehensive characterization of tumor hypoxia across multiple spatial and temporal scales [63].

#### 3.2.3 Applications in Tumor Imaging and Therapy

Conventional theranostic agents often lack tumor‐selective activation, causing systemic toxicity. Hypoxia‐responsive AIE nanoplatforms address this by combining stimuli‐triggered accumulation with multimodal signal amplification. For instance, the metal–organic framework (MOF)‐based system A‐NUiO@DCDA@ZIF‐Cu exemplifies this strategy: under hypoxic conditions (pO_2_ < 10 mmHg), its shell decomposes to release ROS‐generating AIEgens (quantum yield: 20.6%) while providing dual MRI (*r*_1_ = 8.7 mM^−1^s^−1^) and fluorescence guidance. This system achieves 89% tumor growth inhibition in vivo [64]. Similarly, red blood cell membrane‐camouflaged M@AP nanoparticles extend circulation half‐life to 11.2 h (vs. 2.3 h for PEGylated controls) and induce ICD via ROS‐mediated antigen release, synergizing with anti‐PD‐1 therapy to suppress metastasis by 73%.

##### 3.2.3.1 CDT Applications

CDT is an antitumor treatment method that generates •OH via Fenton or Fenton‐like reactions triggered by specific metal ions (such as Fe^2+^, Mn^2+^, Cu^+^, etc.) in the TME. Ning et al. developed a platelet vesicle‐encapsulated AIE photosensitizer TBP‐2 that rapidly degrades and releases copper ions under acidic conditions. Subsequently, it can consume GSH and produce hydroxyl radicals under light irradiation, thereby inducing tumor cell copper death (Cuproptosis) [65]. Zhang et al. designed a system containing AIE photosensitizers and SO_2_ prodrugs (PSAB) that can enhance PDT effects by consuming GSH and effectively inhibit tumor recurrence and metastasis [66].

Compared to pure CDT reagents, these redox‐responsive AIE materials demonstrate significant advantages in treatment targeting, real‐time monitoring of effects, and side effect control.

##### 3.2.3.2 PDT and PTT Combination Therapies

The combination of PDT and PTT, inducing both oxidative stress and hyperthermia simultaneously in cancer cells, offers synergistic advantages. Wen et al. developed AIE dots with broad absorption in the visible region, bright NIR fluorescence emission, and high ROS generation capabilities. These dots demonstrated excellent fluorescence‐photoacoustic imaging‐guided PDT‐PTT synergistic therapy, effectively eliminating tumors with just one injection and irradiation [67]. Khandare et al. developed self‐navigating polymeric prodrug micelles containing two AIE photosensitizers and a reduction‐sensitive paclitaxel prodrug [68]. This system promotes cellular uptake through photochemical internalization and provides precise guidance for PDT through ratiometric fluorescence monitoring. Zhang et al. developed AIE photosensitizers (TPS‐2) with tunable organelle specificity by modulating hydrophobic *π*‐bridges and zwitterionic functional groups. Membrane‐targeting TPS‐2 induces cell death and membrane rupture through PDT, promoting antigen release and immune cell activation to enhance anti‐tumor immunity [69].

#### 3.2.4 Key Challenges and Solution Strategies

A series of hypoxia and redox‐responsive AIE theranostic materials with diverse design strategies have been developed for tumor diagnosis and treatment, which can be mainly classified into four categories: nitro‐reduction response, GSH‐mediated response, ROS regulation, and metal ion reduction response. The detailed design principles, response mechanisms, and representative research systems of these AIE materials are systematically summarized in Table 3. Despite the prominent application potential of these responsive AIE materials, their further clinical transformation still confronts many inherent limitations.

**TABLE 3:** Summary of hypoxia‐ and redox‐responsive AIE materials for tumor theranostics.

Design principle	Response mechanism	Representative systems	References
Nitro‐reduction	Utilize nitro groups as responsive sites for specific recognition and reduction by NTR overexpressed in hypoxic tumor niches	TTVBA, Biotin‐TTVBA DN1–DN6	[50, 51]
GSH‐mediated response	Modulate disulfide bonds in AIE skeletons to achieve specific response to high intracellular GSH in tumor cells	MBTB‐PA AIE/SO_2_ nanoplatform	[53, 54]
ROS regulation	Construct D–*π*–A molecular frameworks to regulate intersystem crossing (ISC), and establish Type I/Type II ROS generation pathways for different oxygen levels	2TT‐2BBTD NPs DPTPzIr	[56, 57]
Metal ion reduction	Fabricate AIE probes targeting reductive metal ions (Fe^2+^, Cu^+^) enriched in the tumor microenvironment	TPE‐FE Cu^+^‐responsive probe	[60, 65]

Tumor microenvironment heterogeneity manifests three key challenges for nitroreductase imaging probes: coexisting hypoxic regions (<5 mmHg) and normoxic areas (>20 mmHg) within 5 mm tumors [70, 71], dynamic oxygen fluctuations with up to 40 mmHg variations within 6 hours caused by abnormal tumor vasculature [72], and the mismatch between the Michaelis constant of nitroreductase probes (Km ≈ 50 μM) and endogenous enzyme concentrations ranging from 2 to 200 μM in tumor tissues [73]. Optical performance limitations include the dramatic quantum yield decrease of traditional TPE derivatives from 60% in solid state to <1% when redshifted to NIR‐II region, self‐absorption issues caused by small Stokes shifts (<50 nm) [74], and the fundamental contradiction between oxygen‐dependent Type‐II photosensitizers and hypoxia‐responsive mechanisms [75]. Biological delivery is hindered by physical barriers (diffusion coefficients <10 μm^2^/s for 50–100 nm particles in tumor stroma), physiological barriers (85% lysosomal trapping at pH 4.5–5.0), and metabolic barriers (70%–90% hepatic sequestration of hydrophobic AIEgens) [76]. Some exploratory molecular designs show improved performance in preclinical tests and provide tentative optimization routes; relevant design ideas may assist follow‐up lab research toward translational exploration [77, 78].

### 3.3 Thermo‐Sensitive AIE Materials in Tumor Applications

Temperature is an important physical parameter for cellular activities, with changes potentially originating from biochemical processes such as cell division, enzymatic reactions, pathological states, or responses to external stimuli [79]. Cancer cells typically have higher temperatures than normal cells, closely related to their higher metabolic rates [80]. Temperature changes are not only related to cancer progression but also closely associated with cancer responses to treatment [81]. Therefore, developing nanothermometers to monitor cellular and tissue temperature changes is crucial for identifying actively proliferating tumor regions.

#### 3.3.1 Design Strategies for Thermo‐Sensitive AIE Materials

##### 3.3.1.1 Lower Critical Solution Temperature Control

Lower critical solution temperature (LCST) control of thermo‐sensitive polymers can induce AIE fluorescence increase under artificial hyperthermia conditions in mouse tumor models (40 °C–43 °C), thereby enhancing fluorescence signals. Poly(N‐isopropylacrylamide) (PNIPAM) is the most widely used thermo‐sensitive matrix, with its LCST mainly adjusted through molecular weight control, chemical modification, and introduction of functional comonomers. The LCST phase transition of PNIPAM originates from temperature‐dependent breakage of intermolecular hydrogen bonds between amide side groups and surrounding water molecules. At temperatures below LCST, extensive hydrogen bonding maintains polymer hydration and molecular dispersion; heating above critical temperature disrupts hydrogen bonding, leaving hydrophobic isopropyl groups to dominate intramolecular aggregation. Polymer chain length and comonomer hydrophilic/hydrophobic substitution synergistically regulate final LCST value rather than following a simple “short chain ↓LCST, long chain ↑LCST” rule, and biological media salinity or serum protein adsorption also shifts actual in vivo transition temperature away from in vitro calibrated LCST. Additionally, LCST can be precisely regulated by introducing hydrophilic groups (such as hydroxyethyl methacrylate or polyethylene glycol) or hydrophobic groups (such as methyl, phenyl) into the PNIPAM main chain [82].

**FIGURE 9:**
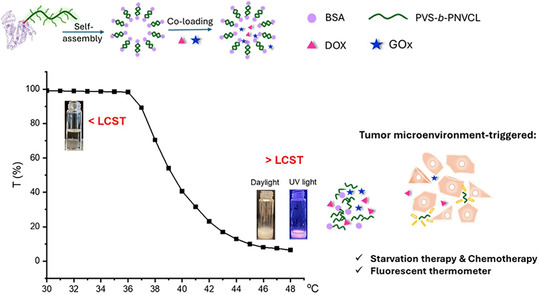
Bovine serum albumin (BSA) and lipid‐based thermoresponsive block copolymers can self‐assemble into vesicular compartments for the codelivery of GOx and DOX. By combining a chemotherapy starvation strategy, they exhibit high drug loading and significant anticancer activity. The LCST of the bioconjugate is adjusted to around 40 °C by incorporating hydrophilic BSA blocks, thereby promoting its targeted drug delivery to tumor cells. These bioconjugates display enhanced intracellular fluorescence intensity with increasing temperature, attributed to the aggregation‐triggered emission of unconventional chromophore components within poly (vinyl caprolactam) (PNVCL). Reproduced with permission [84]. Copyright 2024, American Chemical Society.

Research shows that PNIPAM‐TPE copolymer materials with an LCST of 42 °C can trigger polymer dehydration and contraction through local photothermal treatment temperature elevation, driving TPE molecular aggregation and enhancing fluorescence signals. In breast cancer models, this material's fluorescence intensity increased eight‐fold, produces enhanced fluorescence signals inside transplanted tumors under designated photothermal treatment in mouse experiments [83]. Wang et al. developed an innovative temperature‐sensing theranostic platform utilizing the unconventional AIE properties of poly(vinyl caprolactam) (PNVCL) embedded within BSA‐polymer bioconjugates. The system exploits temperature‐triggered aggregation of PNVCL chromophore components to provide real‐time fluorescence feedback during therapy. Below the LCST (∼40 °C), the bioconjugate maintains a molecularly dispersed state with weak fluorescence; upon reaching tumor thermal therapy temperatures (40 °C–43 °C), PNVCL chains undergo dehydration‐induced aggregation, activating intense AIE fluorescence for treatment monitoring (Figure 9) [84]. This temperature‐responsive AIE behavior enables simultaneous visualization of drug release kinetics and local temperature distribution in tumor cells. The platform integrates GOx‐mediated starvation therapy with DOX chemotherapy, where fluorescence intensity serves as a quantitative indicator of therapeutic efficacy‐brighter emission correlates with enhanced drug liberation and deeper tumor penetration. Notably, the incorporation of hydrophilic BSA segments precisely tunes the LCST to match physiological hyperthermia conditions, shows preferential fluorescence response toward heated tumor sites in preclinical tests.

##### 3.3.1.2 Temperature‐Controlled Chemical Reactions

Temperature‐controlled chemical reactions utilize dynamic covalent bond designs (such as thioether bonds, ester bonds, or boronate ester bonds) to trigger chemical bond breakage or molecular rearrangement in high‐temperature environments, thereby realizing responsive drug release and fluorescence variation under external laser heating in cell and animal tests. These materials’ thermo‐sensitive characteristics provide higher specificity for targeted responses in high‐temperature regions. The thermo‐sensitive AIE material TPE‐CH_3_ based on ester bonds triggers ester hydrolysis at tumor thermal therapy temperatures (>40 °C), releasing fluorophores for breast cancer imaging along with precise delivery of chemotherapeutic drugs [85]. Local high temperatures are typically provided by external PTT (e.g., 808 nm laser heating) or magnetic nanoparticles to achieve temperature control through alternating magnetic fields.

##### 3.3.1.3 Conformational Change Mechanisms

Thermo‐sensitive AIE materials enhance fluorescence signals at specific temperatures through conformational changes in molecular flexible chain segments or D–A structures. Flexible chain segments restrict intramolecular rotation due to chain curling or dehydration collapse at elevated temperatures, significantly boosting fluorescence. The PNIPAM‐TPPEBr nanothermometer designed by Yin et al. copolymerizes TPE derivatives TPPEBr with twisted intramolecular charge transfer (TICT) characteristics and PNIPAM. When temperature rises from 31 °C to 38 °C, PNIPAM chains transform from extended to contracted states, promoting TPPEBr molecular aggregation with fluorescence intensity enhancing by 13.2%/°C. This probe was successfully applied to real‐time monitoring of temperature changes in HeLa cells with a resolution of 0.5 °C [86].

**FIGURE 10:**
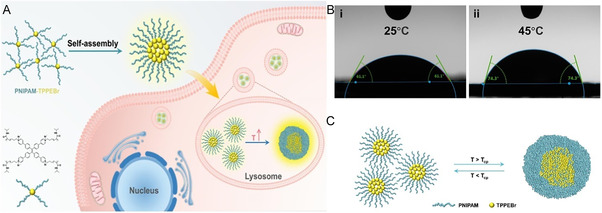
(A) Aggregation‐induced emission‐based “on” polymer nanothermometer for intracellular temperature sensing. (B) Contact angles of PNIPAM‐TPPEBr at (i) 25 °C and (ii) 40 °C. (C) Schematic illustration of the conformational changes of PNIPAM‐TPPEBr with increasing temperature. Reproduced with permission [87]. Copyright 2025, Elsevier Ltd.

D–A molecules undergo conformational rearrangements or electron cloud distribution changes in high‐temperature environments, reducing charge transfer efficiency and causing excitation state energy to release more fluorescence through radiative transitions. The D*–π*–A–D type molecule designed by Zhao et al. uses TPA as an electron donor (D), benzothiadiazole as an acceptor (A), and introduces flexible alkoxy chains to regulate molecular stacking. This material achieves a 40% quantum yield in aggregated states, with fluorescence intensity linearly decreasing in the 25 °C–50 °C range (sensitivity: 1.8%/°C), displays favorable spectral properties for tumor imaging under laboratory experimental conditions (Figure 10) [87].

##### 3.3.1.4 Thermo‐Sensitive Aggregation Effects

Thermo‐sensitive aggregation effects are based on the self‐assembly behavior of thermo‐sensitive molecular fragments (such as fatty acid chains, phenyl ring structures) in high‐temperature environments, utilizing hydrophobic interactions or *π* *–π* stacking effects to promote AIE molecular aggregation, thereby triggering fluorescence signal enhancement. TPE‐FA materials exploit the hydrophobic aggregation effect of fatty acid chains in breast cancer thermal therapy environments (42 °C) to trigger red fluorescence signals, enhancing signal intensity over 10‐fold for deep tumor imaging [88].

TPE‐carbon nanotube (CNT) composite materials in pancreatic cancer models induce material aggregation through near‐infrared PTT (808 nm laser heating to 43 °C), significantly enhancing fluorescence signals while combining the photothermal effect of carbon nanotubes to promote tumor cell ablation. The TPy‐TPE/S‐βCD supramolecular system reported by Kaur et al. involves self‐assembly of cationic TPE derivatives (TPy‐TPE) with sulfonated cyclodextrin (S‐βCD) through electrostatic interactions. As temperature rises from 25 °C to 45 °C, the assembly's crosslinking degree decreases, reducing fluorescence intensity by 60% with the red shift of emission wavelength (Δ*λ *= 20 nm) [89].

#### 3.3.2 Applications in Tumor Imaging

Thermo‐responsive AIE materials utilize metabolically active tumor‐normal tissue temperature differences (2 °C–5 °C) for precise imaging. LCST‐optimized probes (phase transition temperature 40 °C–43 °C) combined with Arg‐Gly‐Asp (RGD) peptide functionalization achieve selective tumor activation with tumor‐background ratios reaching 8:1 [90]. However, low metabolic tumors or small lesions (<5 mm) show insufficient temperature differences (<1 °C) limiting probe activation effects. To overcome this limitation, dual‐targeting strategies combining thermal responsiveness with folate receptor binding reduce nontargeted activation by 72%, while NIR‐II probes (*λ*_em_ = 1300 nm) enhance detection sensitivity for tiny lesions through improved tissue penetration [91].

Based on successful single‐modality imaging applications, dual‐modal systems further achieve dynamic evaluation of therapeutic effects. TPE‐Temp/PTT systems show a strong linear relationship between fluorescence intensity and local temperature (*R* ^2^ = 0.96) as well as residual tumor burden (area under the curve (AUC) = 0.91), providing quantitative indicators for treatment feedback [92]. NIR‐II liposomes (TTS‐2F Lip) combining PDT with fluorescence imaging achieve 85% tumor regression rates under 808 nm light irradiation [93]. A key challenge, however, is overheating of normal tissues (>43 °C) during PTT, which increases background noise by 32%. Spatial focusing laser systems (beam diameter < 2 mm) and ratiometric probe applications effectively address this issue, achieving 89% accuracy in distinguishing tumors (Δ*T* > 2 °C) from inflammatory tissues (Δ*T* < 1 °C) [91].

Given the depth limitations of fluorescence imaging and insufficient specificity of single MRI/CT, multimodal integration becomes a development trend for deep tumor diagnosis. Zhou et al. developed dual‐modal probes that enhance fluorescence signals four‐fold (SNR = 18.3) and T_1_‐weighted MRI signals 2.5‐fold (ΔSNR = 12.7) in 4T1 breast tumors, achieving effective detection of 0.8 mm^3^ lesions [94]. NIR‐II/photoacoustic imaging (PAI) liposomes achieve 421 μm imaging depth in gliomas with three‐fold improved detection sensitivity for 1.5 mm lesions compared to traditional NIR‐I probes [95].

#### 3.3.3 Applications in Tumor Therapy

##### 3.3.3.1 PTT Applications

**FIGURE 11:**
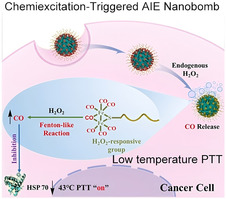
The upregulation of heat shock protein (HSP) expression damages the therapeutic effect of PTT. Inhibiting HSP repair is crucial to improving the efficiency of low‐temperature PTT. The AIE nanobomb PBPTV@mPEG (CO) can be triggered by overexpressed peroxides in the tumor microenvironment, releasing CO to inhibit the upregulation of HSP expression during low‐temperature PTT, thereby enhancing antitumor efficacy. Reproduced with permission [96]. Copyright 2022, Wiley‐VCH GmbH.

Thermosensitive AIE materials demonstrate significant potential in PTT due to their unique temperature‐responsive properties. For instance, the PNIPAM‐TPE system undergoes a temperature‐dependent phase transition above 42 °C, where molecular aggregation enhances its photothermal conversion efficiency by ≈35%. Recent advancements in this field are particularly evident in the development of various novel materials: NIR‐II responsive nanobombs, which address thermal resistance through controlled CO release, reducing heat shock protein 70 (HSP70) expression by 70% and significantly enhancing treatment efficacy at a mild temperature of 45 °C (Figure 11) [96]. Furthermore, the more extreme‐performing NIR⊂TPE‐EA Förster resonance energy transfer (FRET) system can even achieve a localized hyperthermia of 119 °C. With a photothermal conversion efficiency of 37.8%, it enables rapid tumor ablation while minimizing damage to surrounding healthy tissues [97]. For thermo‐sensitive AIE materials targeting organelles to boost therapy, Zou et al. developed lysosome‐targeted AIEgen QM‐DMAC (viscosity‐responsive, thermo‐sensitive); it accumulates in lysosomes (Pearson's coefficient = 0.89), generates ROS to induce lysosomal rupture via “lysosome hijacking” (disrupting pH, restoring radiotherapy sensitivity), and enabled 93% tumor inhibition in A549 models (QM‐DMAC‐PTT + radiotherapy) to overcome resistance [98].

##### 3.3.3.2 Drug Delivery Applications

Thermo‐responsive AIE materials have emerged as promising drug delivery systems for tumor treatment, combining precise temperature‐controlled release with real‐time monitoring capabilities. The PNIPAM‐TPE‐DOX nanocarrier demonstrates temperature‐triggered drug release in breast cancer models. When local temperature reaches 42 °C, the PNIPAM shell undergoes a phase transition, rapidly releasing DOX while enhancing TPE fluorescence for release monitoring. This system achieves four‐fold higher tumor accumulation compared to normal tissues with 78% tumor inhibition rates and reduced systemic toxicity. Recent advances include protein–polymer bioconjugates (PVS‐b‐PNVCL/BSA) for codelivery of glucose GOx and DOX, combining chemotherapy with starvation therapy. The incorporation of BSA adjusts the LCST to ≈40 °C, optimizing tumor‐specific release [99].

#### 3.3.4 Key Challenges and Solution Strategies

Thermo‐sensitive AIE materials are mainly categorized into four types based on working mechanisms: LCST phase transition, temperature‐controlled chemical reactions, temperature‐induced conformational transformation and thermo‐responsive aggregation. Table 4 summarizes their design principles, response mechanisms, and representative research systems. Despite promising application prospects, these materials still face various practical challenges.

**TABLE 4:** Summary of thermo‐sensitive AIE materials for tumor theranostics.

Design principle	Response mechanism	Representative systems	References
LCST	LCST of thermo‐sensitive polymers to regulate molecular aggregation state for reversible transition between dispersed and aggregated states via temperature control	PNIPAM‐TPE PNVCL/BSA PVS‐b‐PNVCL/BSA	[83, 84, 99]
Temperature‐controlled chemical reactions	Modulate dynamic covalent bonds (thioether, ester, boronate ester bonds) to achieve temperature‐triggered bond breakage or molecular rearrangement	TPE‐CH_3_	[85]
Conformational change mechanisms	Construct molecular structures with flexible chain segments or D–A architectures to achieve temperature‐dependent fluorescence modulation via conformational changes	PNIPAM‐TPPEBr D*–π*–A–D QM‐DMAC	[86, 87, 98]
Thermo‐sensitive aggregation effects	Fabricate molecular systems with thermo‐sensitive fragments to promote AIE molecular aggregation via hydrophobic interactions or *π* *–π* stacking under high temperature	TPE‐CNT TPy‐TPE/S‐βCD	[88, 89]

The core scientific issue being an incomplete understanding of their thermal response mechanisms at the molecular level. Recent studies reveal critical knowledge gaps in the structure–activity relationship between the TICT of AIE molecules and their temperature sensitivity [100]. Molecular dynamics simulations show that when the temperature exceeds the LCST, the intramolecular rotational energy barrier significantly decreases. This unclear molecular mechanism directly leads to multiscale heat transfer dynamics issues. In tumor tissues, which exhibit significant spatial heterogeneity in thermal diffusivity (0.1–0.5 W/m·K), conventional thermal therapy can produce temperature gradients as steep as 15 °C/cm. To address this bottleneck, researchers have developed quantum dot‐AIE hybrid systems, achieving a temperature resolution of 0.1 °C through precise modulation of FRET efficiency. However, clinical translation still faces profound contradictions, most notably the mismatch between thermal therapy and imaging parameters: the deviation between the optimal therapeutic temperature window (41 °C–43 °C) and the optimal imaging sensitivity range (35 °C–39 °C) results in a 35% efficacy difference [101].

To tackle these challenges, cutting‐edge research is advancing along three key directions: supramolecular temperature sensors, in vivo self‐calibration systems, and AI‐assisted design. Particularly noteworthy is the innovative design of metabolically programmable materials, which reduce liver accumulation by 76% through pH/GSH dual‐responsive bonds, while adaptive optical systems combining computational optics and deep learning enhance tissue penetration depth to 8 cm. The field is currently at a critical juncture, transitioning from empirical exploration to rational design, necessitating the establishment of new theoretical frameworks and technical standards to address systemic challenges spanning molecular mechanisms to clinical applications [102].

### 3.4 Applications for Enzyme‐Responsive AIE Materials in Tumor Applications

Tumors exhibit overexpression of various specific enzymes in their microenvironment, which are closely related to biological behaviors such as tumor invasion, metastasis, metabolic regulation, and immune evasion. Based on this characteristic, researchers have developed a series of enzyme‐responsive AIE materials for precise tumor diagnosis and treatment. Currently, MMPs and ALP, which are widely overexpressed in various tumors, are the most extensively studied enzyme targets, while cathepsins and lipases show stronger specificity in certain tumor types [84].

#### 3.4.1 Design Strategies for Enzyme‐Responsive AIE Materials

##### 3.4.1.1 MMP Response Mechanisms

**FIGURE 12:**
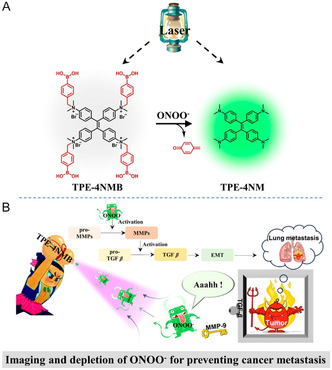
Schematic illustration of TPE‐4NMB for (A) detection and (B) depletion of ONOO^−^ to prevent cancer metastasis mediated by MMP‐9 and TGF‐β. Reproduced with permission [109]. Reproduced with permission [104]. Copyright 2024, American Chemical Society.

Matrix MMPs are key enzymes involved in degrading the extracellular matrix and basement membranes, playing a critical role in tumor invasion and metastasis. Notably, MMP‐2 and MMP‐9 are significantly overexpressed at the invasive fronts and core regions of cancers such as breast, pancreatic, and colorectal tumors. Their ability to facilitate tumor cell migration and invasion makes them important targets for research on metastasis and the development of MMP‐responsive fluorescent probes. MMP‐targeted AIE materials typically incorporate specific peptide substrates (e.g., PLGVR or GPLGVRG) that are cleaved by MMPs, triggering fluorescence emission. For example, TPE‐PLGVR precursor carries hydrophilic peptide GPLGVR moiety conjugated onto hydrophobic AIE core to achieve homogeneous aqueous dispersion with quenched fluorescence. After site‐specific hydrolysis of Gly‐Leu peptide linkage catalyzed by overexpressed MMP‐2 at tumor invasive front, detached hydrophilic peptide fragments dissolve in aqueous phase and leave bare hydrophobic TPE skeletons to spontaneously aggregate and switch on AIE fluorescence for tumor visualization. Besides ECM degradation, activated MMP‐2 modulates TGF‐β‐associated epithelial‐mesenchymal transition (EMT) to accelerate tumor metastasis [103]. Zhang et al. developed a theranostic nanoprobe with dual functionality: MMP‐responsive imaging and modulation of the TME. Under the action of peroxynitrite (ONOO^−^), this probe transforms into TPE‐4 NM^−^, exhibiting strong AIE fluorescence (120‐fold increase) while also reducing ONOO^−^ levels. Importantly, it inhibits 4T1 cell migration and downregulates MMP‐9 and transforms growth factor‐β (TGF‐β) into residual tumors, suppresses EMT and significantly reduces lung metastasis risk in postoperative breast cancer models (Figure 12) [104]. Beyond the aforementioned peptide‐triggered AIE switching, optimized AIE‐peptide conjugates have been developed for tumor theranostics. One typical design tethers MMP‐cleavable peptides and hydrophilic fragments onto hydrophobic AIE skeletons. Intact precursors disperse weakly emissive under physiological conditions; after MMP digestion at invasive tumor sites, exposed hydrophobic cores aggregate to produce high‐contrast fluorescence (T/N > 10:1) for tiny residual lung tumor delineation, meanwhile blocking metastasis‐associated kinases to realize imaging‐guided chemotherapy [105].

##### 3.4.1.2 ALP Response Mechanisms

**FIGURE 13:**
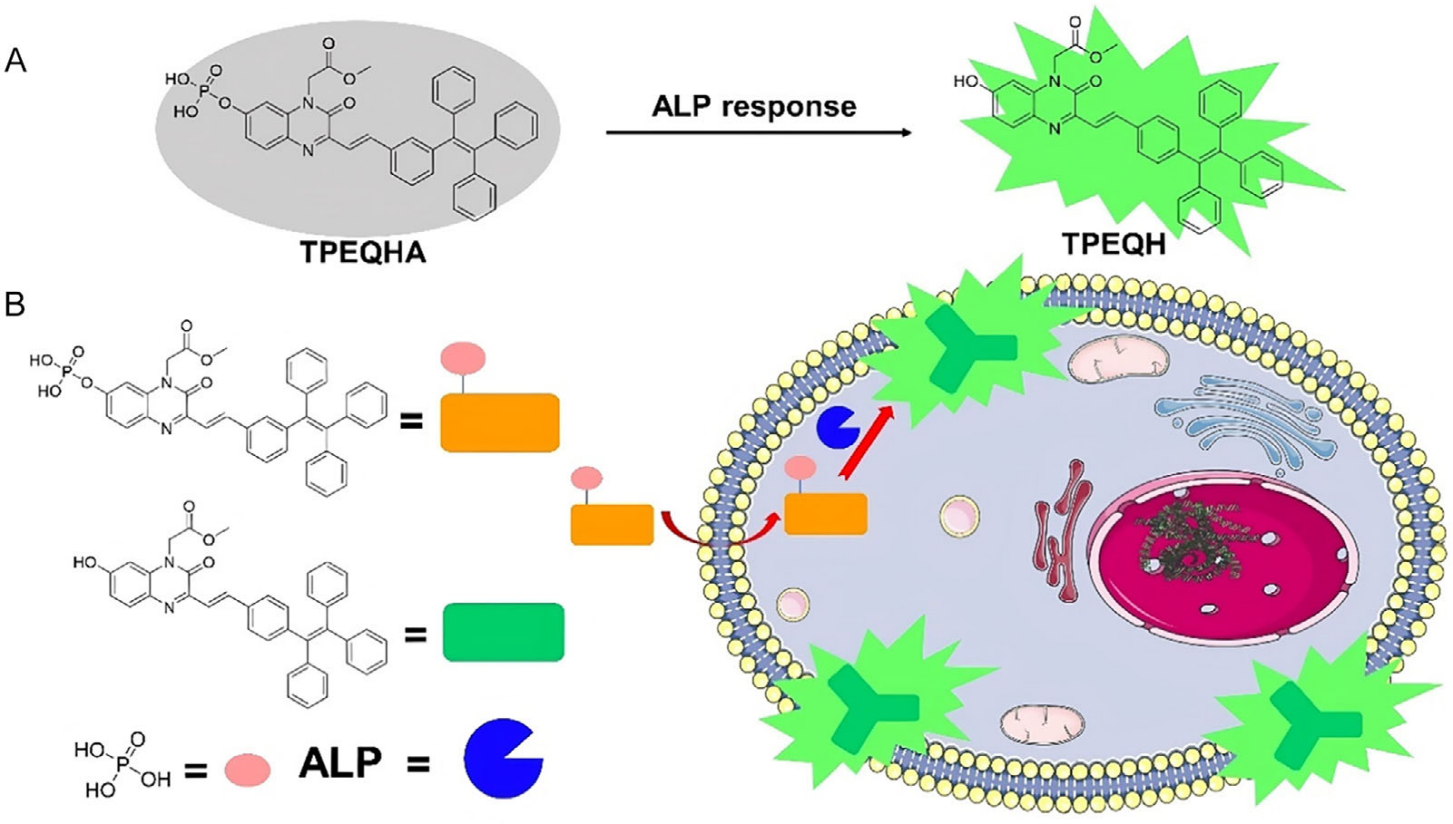
(A) Water‐soluble TPEQHA is hydrolyzed by ALP to become the lipid‐soluble TPEQH, which aggregates in aqueous solution to induce fluorescence. (B) In tumor cells, TPEQHA is hydrolyzed on the cell membrane by ALP and aggregates to induce fluorescence. Reproduced with permission [107]. Copyright 2020, Elsevier Ltd.

Alkaline phosphatase (ALP) is a metabolic enzyme highly expressed in tumors such as liver cancer and osteosarcoma, where its activity correlates with tumor grade and aggressiveness. Membrane‐tethered ALP overexpressed on hepatocarcinoma and osteosarcoma cell surfaces catalyzes selective dephosphorylation of phosphate‐capped AIE precursors. The negatively charged hydrophilic phosphate group is enzymatically cleaved into inorganic phosphate, converting highly water‐soluble phosphorylated AIE into lipophilic parent luminogen; loss of aqueous solubility drives spontaneous aggregation and consequent AIE activation predominantly on tumor cell membrane surface. ALP accelerates malignant progression via regulating intracellular phosphate metabolism and energy homeostasis instead of direct ECM proteolysis. In liver cancer, pTPE probes emit blue fluorescence upon ALP activation, enabling early tumor detection [106]. Recent advances have expanded ALP‐responsive AIE materials into theranostics. Guan et al. developed TPEQH, a phosphate‐ester‐modified AIE probe that precipitates as bright fluorescent aggregates in ALP‐rich tumor cells, improving imaging precision while enabling targeted therapy (Figure 13) [107]. Apart from phosphate‐capped AIE for hepatoma detection, novel ALP‐activated fluorophores expand to osteosarcoma treatment. Phosphorylated AIE precursors stay soluble and dim under normal environments; membrane‐bound ALP on osteosarcoma removes phosphate shielding to trigger fluorophore aggregation, produces ROS and suppresses tumor proliferation within tested orthotopic osteosarcoma mouse groups [108].

##### 3.4.1.3 Lipase Response Mechanisms

**FIGURE 14:**
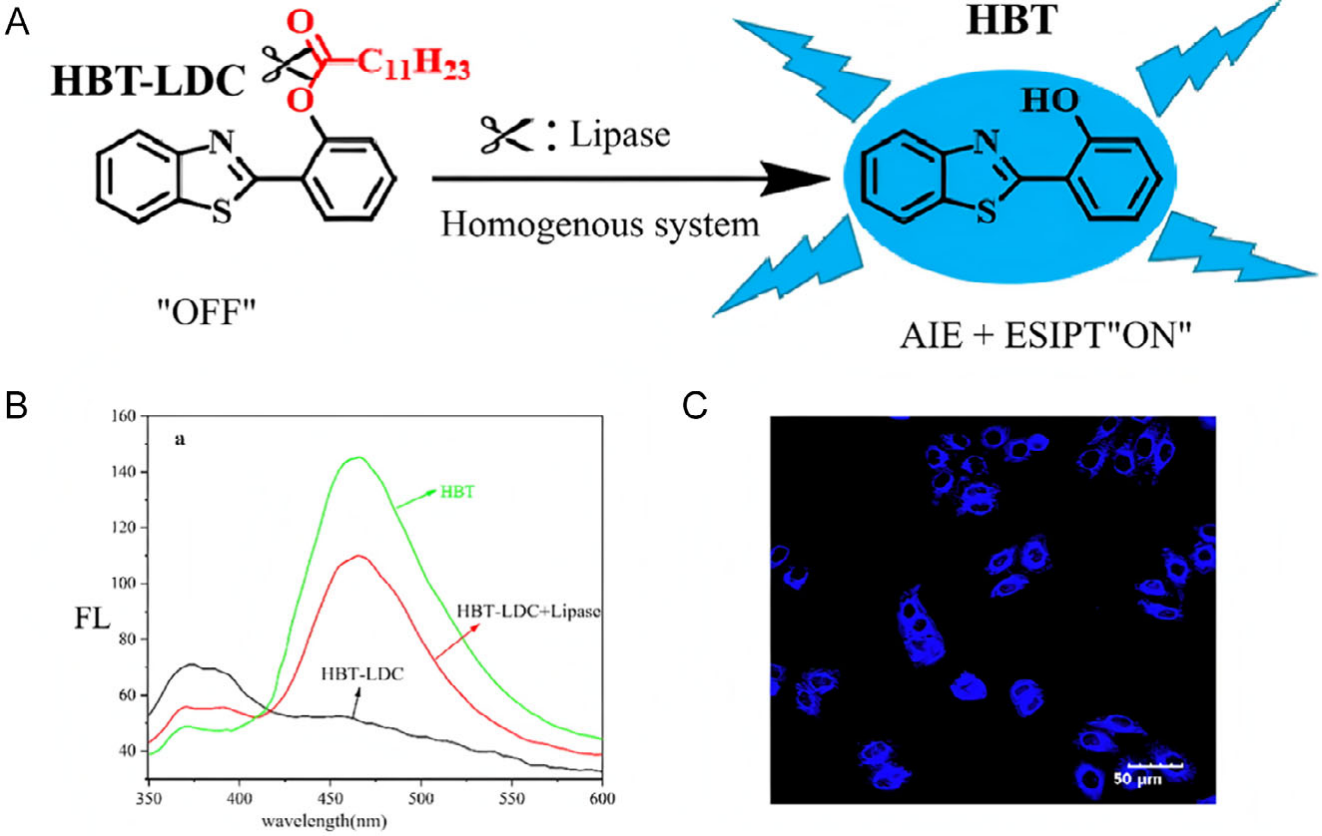
(A) The interaction process between pancreatic lipase and the HBT‐LDC probe. (B) Fluorescence spectra of HBT‐LDC, HBT, and HBT‐LDC + lipase under 330 nm excitation. (C) HeLa cells pretreated with exogenous lipase for 2 h and further incubated with HBT‐LDC for 30 min at 37 °C. Reproduced with permission [109]. Copyright 2022, Elsevier Inc.

Tumor‐overexpressed lipase features canonical interfacial activation property: the enzyme's hydrophobic lid domain undergoes conformational opening exclusively at oil–water biphasic interface to expose active catalytic pocket and gain full hydrolysis activity. After enzymatic cleavage of ester‐linked hydrophilic side chains on AIE probes, residual hydrophobic fluorophore backbones aggregate at lipid–water interphase to turn on AIE signals, endowing high selectivity toward lipid‐rich hepatic and pancreatic tumor tissues. Lipase‐responsive AIE materials are typically designed by conjugating AIE fluorophores with enzyme‐cleavable groups (e.g., esters or amides), which undergo hydrolysis‐induced structural changes to trigger fluorescence. A common strategy uses fatty acid/ester substrates that, upon lipase cleavage, expose hydrophobic fragments to induce aggregation and luminescence. A major challenge in lipase probe development lies in the interfacial nature of lipase reactions (between hydrophilic enzymes and hydrophobic substrates). Zhang et al. developed HBT‐LDC, a benzimidazolone‐based probe combining AIE and excited‐state intramolecular proton transfer properties for lipase detection. It exhibits high selectivity (detection limit: 0.01 mg/mL), no self‐quenching, and large Stokes shifts, enabling lipase activity imaging in HeLa cells (Figure 14) [109]. Shi et al. designed a glutamic acid‐functionalized TPE probe (S1) that achieves rapid (7 min), ultrasensitive lipase detection (limit: 0.13 U/L) at oil–water interfaces, with a low Km (4.23 μM) indicating strong lipase affinity [110]. Benefiting from overexpressed lipase in lipid‐rich tumors, ester‐modified AIE probes target pancreatic and fatty hepatocellular carcinoma. Ester‐functionalized precursors remain nonfluorescent and uniformly dispersed in body fluids; tumor lipase at oil–water interfaces selectively hydrolyzes ester side chains to unmask hydrophobic cores and turn on luminescence, which distinguishes two kinds of tissue samples under specified in vitro and mouse experimental conditions [11].

#### 3.4.2 Applications in Tumor Imaging

Enzyme‐responsive AIE materials have revolutionized tumor imaging through their specific enzymatic recognition and signal amplification capabilities. Single‐modality imaging systems designed for high‐contrast optical imaging target tumor‐associated enzymes like MMPs and cathepsins. For example, a cathepsin B‐responsive TPE‐RR probe demonstrates exceptional specificity in breast cancer models, generating red fluorescence upon enzymatic cleavage while enabling combined chemo‐theranostics. Trypsin‐responsive lysine‐peptide probes provide precise boundary delineation in pancreatic tumors. While offering excellent sensitivity, single‐modality systems face challenges with tissue penetration depth and potential false positives from inflammatory enzyme activity [111].

Dual‐modality imaging systems address depth limitations by combining optical imaging with other techniques. The MMP‐2‐responsive NIR‐II probe achieves 8 mm tissue penetration in pancreatic models with six‐fold signal enhancement [112], while the DPP‐P@Gd‐TPE system merges ALP‐activated fluorescence with MRI contrast for liver cancer imaging. Such designs leverage optical sensitivity and the spatial resolution of complementary modalities, significantly improving diagnostic accuracy for deep‐seated tumors [103].

Advanced multimodal theranostic systems integrate imaging with therapeutic functions. The CD73‐responsive NIR‐II probe CPT‐S‐PEG enables real‐time immunotherapy monitoring in lymphoma models when combined with anti‐programmed death‐ligand 1 therapy [113]. Another innovative design uses temperature‐sensitive ALP probes (TPE‐CN‐pho) that enhance signal output during PTT (40 °C–45 °C), creating a positive feedback loop for treatment monitoring [114]. These systems often employ ratio imaging techniques (e.g., MMP/ALP activity ratios) and targeted delivery to minimize off‐target effects.

#### 3.4.3 Applications for Tumor Therapy

##### 3.4.3.1 PTT Applications

**FIGURE 15:**
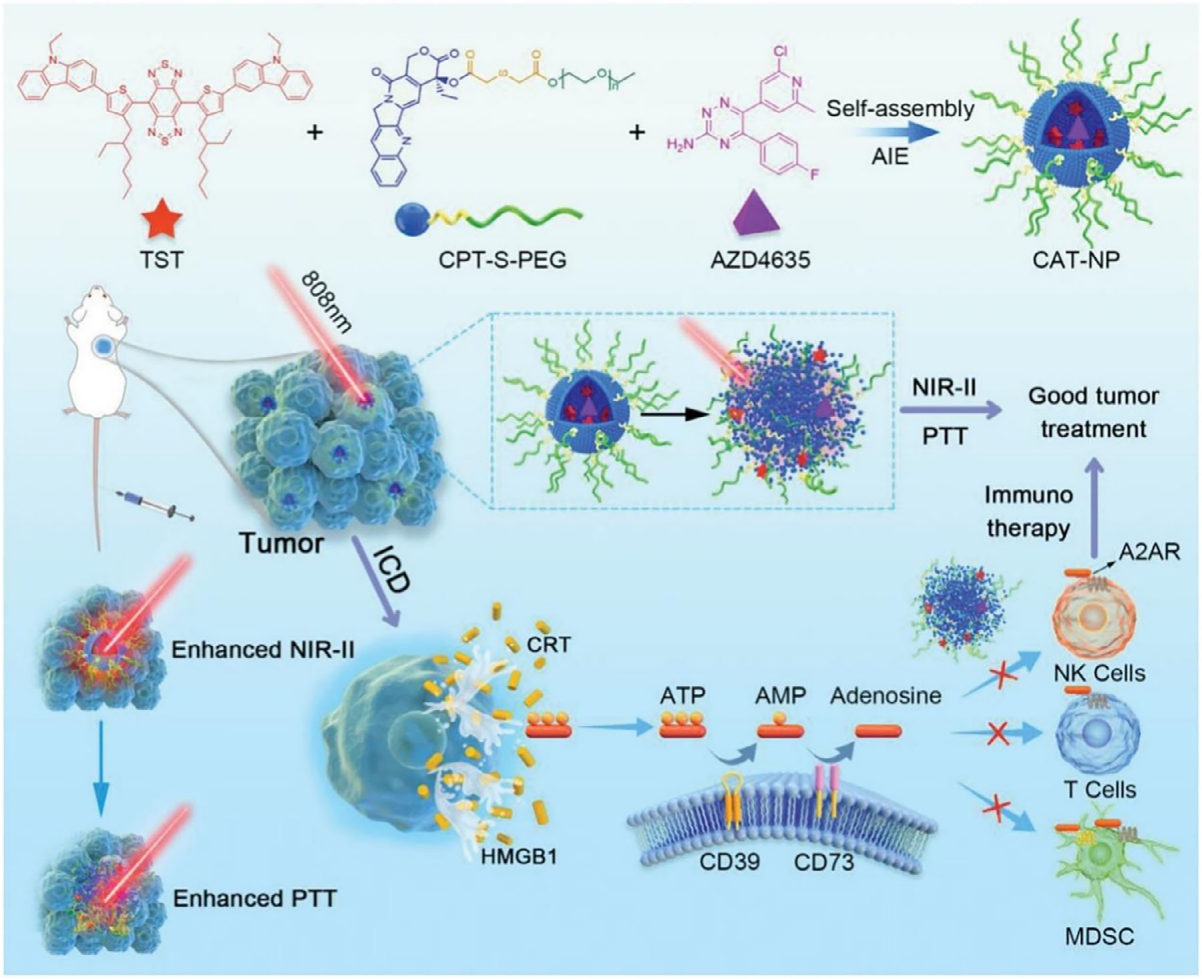
CAT‐NP is fabricated by coassembling TST, CPT‐S‐PEG, and AZD4635 via nanoprecipitation. After intravenous injection, CAT‐NP passively accumulates in tumors through the EPR effect. Intact CAT‐NP affords prominent AIE‐enhanced NIR‐II imaging, while nanoparticle disassembly improves PTT efficacy. PTT induces tumor ICD and releases multiple immunogenic molecules including ATP. Tumor‐derived ATP is further converted into immunosuppressive adenosine through the CD39‐CD73‐A2AR pathway. The released AZD4635 blocks adenosine‐A2AR binding, relieving immune suppression, and augmenting antitumor immunotherapy. Reproduced with permission [113]. Copyright 2022, Advanced Science published by Wiley‐VCH GmbH.

PTT has emerged as a promising tumor treatment approach due to its minimally invasive nature and therapeutic efficacy. Wang et al. developed NIR‐II emitting TST nanoparticles coassembled with camptothecin prodrug (CPT‐S‐PEG) and immune checkpoint inhibitor AZD4635, which exhibited redox‐responsive decomposition in cancer cells. The released TST enhanced photothermal conversion while PTT‐induced ICD triggered ATP release. Simultaneously, AZD4635 blocks the CD39‐CD73‐adenosine 2A receptor (A2AR) pathway, preventing ATP conversion to immunosuppressive adenosine and thereby achieving combined photothermal‐chemotherapy‐immunotherapy effects (Figure 15) [113]. Zhang et al. demonstrated the versatility of PEGylated AIE molecules for dual ATP/H_2_S sensing and PDT, with TPE‐PEG160 enabling cellular microenvironment monitoring and TPE‐PEG750 serving as an effective photosensitizer [114].

##### 3.4.3.2 PDT Applications

Enzyme‐responsive AIE materials offer unique advantages in PDT by responding specifically to highly expressed enzymes in tumors, enabling precise photosensitizer release and ROS generation [115]. The ALP‐responsive AIE photosensitive probe (TPE‐Py) significantly improves ROS generation efficiency through ALP enzymatic cleavage‐triggered molecular aggregation, while monitoring treatment areas in real time via fluorescence [116]. The Biotin‐conjugated Ru (II) probe, through caspase‐3 and lactate dehydrogenase dual‐enzyme synergistic response, induces ROS generation and NADH consumption in small cell lung cancer models through 460 nm light irradiation, disrupting redox homeostasis in A549 cells and activating mitochondrial apoptosis pathways [117]. For enzyme‐responsive AIE photosensitizers leveraging organelle cascade targeting to enhance PDT, Mai et al. designed amphiphilic AIE photosensitizer SgTBB (mitochondria‐to‐nuclei cascade targeting) that generates type I (•OH/O_2_^−^•) and type II (^1^O_2_, QY = 0.63) ROS; in vitro it first accumulates in mitochondria (Pearson's coefficient = 0.87) then translocates to nuclei postlight activation, and in 4T1 models, SgTBB‐mediated PDT achieved 91% tumor inhibition and induced ICD (82% calreticulin exposure) to boost anti‐tumor immunity [118].

Enzyme‐responsive AIE materials in PTT trigger photothermal effects through enzymatic cleavage, significantly enhancing photothermal conversion efficiency and enabling dynamic treatment monitoring via fluorescence. The lipase‐responsive photothermal probe (TPE‐BBT) can suppress the DNA repair capability of cancer stem cells through photothermal effects, effectively preventing cancer recurrence and metastasis after radiotherapy [119]. In pancreatic cancer treatment, the MMP‐2 and Cathepsin B dual‐enzyme synergistic responsive photothermal probe (TPE‐M@Au‐CatB) precisely controls tumor temperature to 45 °C–50 °C through a two‐step triggering mechanism, while dynamic fluorescence signal monitoring avoids normal tissue damage from heat diffusion [120].

##### 3.4.3.3 Chemotherapy Applications

In chemotherapy, enzyme‐responsive AIE materials precisely target specifically overexpressed enzymes in tumors, promoting localized release of chemotherapeutic drugs, significantly improving treatment efficiency and reducing systemic side effects. The B‐cell lymphoma 2 (Bcl‐2)‐responsive AIE chemotherapy probe (TPEDCC) releases DOX through Bcl‐2 enzymatic cleavage, significantly increasing drug accumulation in tumor sites (4 times higher than normal tissues), while enabling dynamic monitoring of the drug release process via blue fluorescence [121].

The probe combining ALP response with PTT (DOX‐TPE‐ALP‐PTT) releases DOX through ALP enzymatic cleavage while increasing cell membrane permeability through photothermal effects, promoting deep drug penetration and increasing tumor inhibition rates to 93% [122].

#### 3.4.4 Key Challenges and Solution Strategies

Based on different catalytic characteristics of tumor‐related enzymes, enzyme‐responsive AIE theranostic materials can be divided into three main types: MMP‐responsive, ALP‐responsive, and lipase‐responsive systems. Table 5 systematically summarizes their design principles, response mechanisms, and typical research platforms. Although various optimization strategies have been proposed to tackle the above limitations, enzyme‐responsive AIE materials still confront multiple obstacles in clinical translation.

**TABLE 5:** Summary of enzyme‐responsive AIE materials for tumor theranostics.

Design principle	Response mechanism	Representative systems	References
MMP response	Utilize MMP‐cleavable specific peptide substrates (e.g., PLGVR, GPLGVRG) conjugated to hydrophobic AIE cores to achieve enzyme‐triggered AIE fluorescence switching and theranostic functions	TPE‐PLGVR TPE‐4NMB AIE‐peptide conjugates	[103, 104, 105]
ALP response	Modify AIE precursors with phosphate‐capped groups to achieve ALP‐catalyzed dephosphorylation‐triggered AIE activation and theranostic effects	pTPE probes TPEQH Phosphorylated AIE	[106, 107, 108]
Lipase response	Conjugate AIE fluorophores with lipase‐cleavable ester/amide groups to achieve interfacial activation‐triggered AIE fluorescence switching	HBT‐LDC probe S1‐TPE probe Ester‐modified AIE probes	[109, 110, 111]

At the molecular level, the insufficient specificity of enzyme responsiveness stems from the baseline expression of tumor‐associated enzymes (e.g., MMP‐2/9, cathepsin B) in normal tissues and the complex regulation of enzymatic activity [123]. Recent studies reveal that spatiotemporal heterogeneity in enzyme activity within the TME (e.g., significant differences between invasive fronts and necrotic cores) leads to substantial regional variations in AIE probe activation efficiency.

Optical performance limitations pose another major hurdle. Conventional AIEgens are constrained by their excitation/emission wavelengths, which not only restrict tissue penetration depth but also overlap with the absorption spectra of endogenous chromophores (e.g., hemoglobin, melanin), reducing signal‐to‐noise ratios [124]. Furthermore, the pharmacokinetics of AIE materials are closely linked to their surface chemistry—excessive hydrophobicity may lead to rapid clearance by the reticuloendothelial system, while excessive hydrophilic modification can impair enzyme responsiveness.

To address these challenges, cutting‐edge research is advancing on multiple fronts. At the molecular design level, “dual‐lock” strategies (e.g., combining MMP‐2‐cleavable peptides with glutathione‐sensitive moieties) improves tumor/normal signal ratio [125]. In optical optimization, D–A structured NIR‐II AIEgens leverage ICT modulation to achieve deep‐tissue imaging (1050–1350 nm) with large Stokes shifts (>300 nm), minimizing excitation interference [126]. Notably, stimuli‐responsive AIE systems are evolving from single‐enzyme activation to multiparameter regulation. For instance, pH/enzyme dual‐responsive systems.

### 3.5 Externally Triggered AIE Materials in Tumor Applications

External physical stimuli‐including acoustic waves, magnetic fields, electrical currents, and mechanical forces‐offer unique advantages for tumor theranostics through remote controllability, deep tissue penetration, and spatiotemporal precision. Unlike endogenous chemical triggers (pH, enzymes, and redox), these modalities enable on‐demand activation with adjustable intensity and location, minimizing off‐target effects while providing real‐time imaging feedback. This section systematically reviews three major classes of physically responsive AIE systems (acoustic, magnetic, electrical) from material design, precision imaging diagnosis to synergistic tumor therapy, and finally summarizes the core challenges and prospective solution strategies of current externally triggered AIE tumor applications.

#### 3.5.1 Design Strategies for Externally Triggered AIE Materials

The rational molecular modification, supramolecular assembly, and nanoplatform construction are the core design principles of physical stimuli‐responsive AIE materials. Targeting the response characteristics of ultrasound, magnetic field, and electric field, diversified design strategies have been developed to optimize optical performance, stimulus responsiveness, tumor targeting ability and biological safety, laying a material foundation for subsequent imaging diagnosis and tumor treatment.

##### 3.5.1.1 Acoustic (Ultrasound) Responsive AIE Material Design

Recent advances in ultrasound‐responsive AIE materials have focused on three synergistic design strategies: molecular engineering, supramolecular assembly, and nanoplatform construction, which jointly optimize sonodynamic performance, tumor targeting, and biological barrier penetration capability.

At the molecular level, D–A structured AIEgens demonstrate enhanced sonodynamic performance through optimized ICT and improved ROS generation efficiency. For example, the TPA‐Tpy molecule featuring an A–D–A framework with cationic modification increased Type I ROS production (superoxide anion/hydroxyl radical) by 2.3‐fold compared to neutral analogs‐showing favorable oxygen tolerance compared with oxygen‐dependent Type II pathways under hypoxic experimental environments [127]. The cationic pyridinium moiety enables mitochondrial targeting through membrane potential‐driven accumulation, with the TCSVP sonosensitizer (TPA‐pyridinium structure) achieving a relatively high ROS quantum yield of 0.89 under specific ultrasonic parameters in laboratory experiments under 1.0 MHz ultrasound irradiation (1.5 W/cm^2^). Mechanistic studies reveal that ultrasound‐induced cavitation generates transient high‐energy microbubbles that enhance ISC efficiency in AIEgens, promoting triplet state formation essential for ROS generation. Yang et al. developed a Type I AIE photosensitizer (MeOTTMN) with twisted molecular architecture and NIR emission (*λ*_em_ = 820 nm). To address PDT's light penetration limitation, they combined MeOTTMN with an implantable wireless‐charged LED (wLED) that emits 660 nm light. MeOTTMN generates •OH/O_2_^−^• (Type I ROS) under wLED irradiation, avoiding oxygen dependence. In 4T1 breast cancer models, this system achieved 87% tumor inhibition with 12 mm tissue penetration‐3‐fold deeper than conventional PDT [128].

Supramolecular assembly strategies address the dual challenges of tumor targeting and sustained circulation. The AIE/Biotin‐M system elegantly combines salicylaldehyde azine AIE units (photophysical core) with DSPE‐PEG‐Biotin (targeting ligand), achieving 3.2‐fold greater tumor accumulation versus nontargeted formulation through biotin receptor‐mediated endocytosis in 4T1 breast cancer models. Building on this principle, Peng et al. developed ABM‐M micelles with an optimized 3:1 biotin‐to‐mannose ratio, enabling simultaneous targeting of tumor cells (biotin receptors) and tumor‐associated macrophages (mannose receptors). This dual‐targeting strategy increased tumor enrichment 4.8‐fold while altering the immunosuppressive TME—a paradigm shift from conventional single‐target approaches [140].

Nanoplatform innovations leverage biomimetic design principles to overcome biological barriers. Patient‐derived apoptotic mimetic vesicles (AMVs) camouflaged with “eat me” signals (phosphatidylserine) demonstrated 6.5‐fold superior bladder cancer targeting compared to synthetic PEGylated nanoparticles, exploiting tumor‐associated macrophages’ phagocytic machinery. The PAHN system employs a stimulus‐responsive “shell‐core” architecture: the polyethylene glycol shell maintains stealth circulation, while caspase‐3 overexpressed in apoptotic tumor cells cleaves a peptide linker (DEVD sequence), triggering AIEgen aggregation and 40% higher ROS yield localized to dying tumor regions—a self‐amplifying therapeutic cascade [129].

In addition, mechanoresponsive AIE design derived from ultrasound mechanical stimulation further expands the acoustic response system. A bifunctional norbornenone‐based mechanophore couples therapeutic gas release with fluorescence reporting. Under focused ultrasound mechanical stimulation, the mechanically labile C—O bond cleaves, releasing carbon monoxide while generating cyan fluorescence enhancement through altered conjugation pathways, realizing integrated design of treatment and real‐time monitoring [130]. For tumor cell‐specific response, LDL‐encapsulated AIE photosensitizer (TPA‐DPPy) is designed to respond to intracellular mechanical stress during tumor cell apoptosis, realizing real‐time mechanical signal reporting of therapeutic effects [131].

##### 3.5.1.2 Magnetic Field Responsive AIE Material Design

Magnetoresponsive AIE systems typically integrate three core functional modules through rational molecular and nanoscale design: (i) AIE‐active fluorophores (TPE, silole derivatives) with emission tunable from visible (450–650 nm) to near‐infrared (650–900 nm) through donor‐acceptor modifications; (ii) magnetic elements (gadolinium chelates for T_1_‐weighted MRI, superparamagnetic iron oxide for T_2_‐weighted imaging); and (iii) biocompatible linkers/matrices (albumin, polymeric micelles, and mesoporous silica) ensuring colloidal stability and controlled biodistribution. The multimodule integrated design realizes the organic combination of magnetic response imaging and AIE optical performance, breaking the independent working limitation of traditional single‐modal imaging probes.

Wang et al.'s breakthrough design conjugated Gd‐DOTA (clinical MRI contrast agent) with a TPE derivative, where albumin‐induced nanoassembly (NGd‐AAs) produced synergistic enhancements. Molecular aggregation restricts intramolecular rotation of TPE fluorophores and activates AIE fluorescence with a six‐fold quantum yield elevation (0.08 → 0.48). Meanwhile, clustered Gd^3+^ chelates after nanoassembly prolong rotational correlation time (*τ*_r_) matching the Larmor precession frequency of surrounding bulk water molecules, optimizing inner‐sphere water exchange kinetics and yielding a 17‐fold improvement in longitudinal relaxivity (*r*_1_) relative to discrete monomeric Gd‐DOTA. This dual amplification mechanism‐fluorescence via RIM and MRI contrast via cooperative magnetic effects—represents a fundamental advantage of AIE‐based multimodal probes over conventional systems [132].

Building on this principle, Xiao et al. developed OEGMA‐HPMA block copolymers incorporating hydrophilic Gd‐DOTA segments (MRI function) and hydrophobic TPE‐benzophenone (TPBP, fluorescence function) within a single macromolecular architecture. The amphiphilic design enables spontaneous micelle formation (hydrodynamic diameter: 85 nm), providing extended circulation half‐life (*t*_1__/__2_ = 6.8 h vs. 0.3 h for free Gd‐DOTA) and preferential tumor accumulation (12.4% ID/g at 24 h postinjection) [133].

Targeted modification design further optimizes tumor specificity. T7 peptide‐modified AIE/Gd nanoparticles (T7‐AIE‐Gd) exploit transferrin receptor (TfR) overexpression in hepatocellular carcinoma for efficient targeted uptake. Zhang et al.'s BK@AIE NPs adopt bradykinin B2 receptor‐mediated transcytosis design, successfully overcoming the blood–tumor barrier in glioblastoma and significantly improving brain tumor accumulation efficiency while maintaining blood–brain barrier integrity [134].

##### 3.5.1.3 Electrical Stimulus Responsive AIE Material Design

Electroresponsive AIE materials rely on electric field‐regulated molecular photophysical behavior, and three mature and efficient design strategies have been formed: (i) donor–acceptor molecular structure design, where external electric fields modulate ICT efficiency to adjust AIE fluorescence intensity and wavelength; (ii) ionic AIEgen design responsive to field‐induced ion migration, realizing reversible optical signal switching; and (iii) conjugated polymer electrochromic structure design, achieving stable electrical response and optical output. These designs endow AIE materials with millisecond‐level temporal precision and sub‐millimeter spatial control activation characteristics.

Li et al.'s NIR‐II electroresponsive AIEgen (2TT‐m, oC6B) adopts a Janus‐type phenothiazine‐thiadiazoloquinoxaline structure, which has excellent NIR‐II emission performance (1000–1300 nm) and high molar absorptivity (1.12 × 10^4^ M^−1^cm^−1^). Rational molecular structure optimization enables electric field stimulation to significantly enhance fluorescence intensity, realizing ultra‐precise tumor visualization [135]. Ma et al. designed an amphipathic AIE agent TDTMSB with a “3 + 2” cooperation pattern, which integrates NIR‐II emission, Type I/II ROS generation, photothermal performance, and lysosome targeting function through precise molecular regulation, providing a multifunctional electro‐responsive AIE platform for multimodal diagnosis and synergistic therapy [136].

For controllable drug delivery applications, TPE‐based conjugated polymer dots (Pdots) with donor‐acceptor tunable emission architecture (P1–P3 series) are designed to respond to electric field pulses. The electric field triggers structural changes of polymer dots, realizing on‐demand release of loaded paclitaxel, and matches the peak of tumor accumulation to optimize therapeutic efficacy [135]. In addition, AIE‐COF/CuS@Ag composite sensor design combines AIE optical performance with plasmonic enhancement effect, realizing ultra‐sensitive electrochemiluminescence (ECL) response for tumor biomarker detection [133].

#### 3.5.2 Imaging Performance of Externally Triggered AIE Materials for Tumor Diagnosis

Benefiting from the precise external field activation and optimized material design, acoustic, magnetic, and electrical responsive AIE materials have achieved high‐sensitivity and high‐specificity tumor imaging. The multiphysical field responsive imaging modes make up for the defects of single‐modal imaging, and realize deep penetration, real‐time monitoring, and ultra‐early diagnosis of tumors.

##### 3.5.2.1 Ultrasound‐Triggered AIE Tumor Imaging

Ultrasound has the advantages of deep tissue penetration (>10 cm) and nonionization, which can assist AIE materials to realize deep tumor imaging and real‐time treatment monitoring. Different from static fluorescence imaging, ultrasound‐triggered AIE imaging combines mechanical cavitation effect and optical signal output, realizing functional imaging of tumor mechanical microenvironment and therapeutic response.

Mechanically responsive AIE imaging derived from ultrasound stimulation can dynamically feedback TME changes and treatment effects. The norbornenone‐based mechanophore realizes real‐time quantitative imaging of CO therapeutic gas release through ultrasound‐triggered fluorescence enhancement, and the fluorescence intensity has a high linear correlation with CO concentration (*R* ^2^ = 0.91), which can provide accurate dosimetry feedback for tumor gas therapy [130]. The LDL‐encapsulated TPA‐DPPy AIE system can respond to intracellular mechanical stress changes during tumor cell apoptosis, producing 3.2‐fold fluorescence enhancement with 15 nm blue‐shift. This real‐time mechano‐optical signal can distinguish therapy‐responsive tumor cells from resistant populations, realizing dynamic imaging monitoring of PDT treatment effect and guiding personalized dose adjustment [131].

##### 3.5.2.2 Magnetic Field‐Triggered AIE Dual‐Modal Tumor Imaging

Magnetoresponsive AIE materials integrate the high molecular sensitivity of fluorescence imaging and the high spatial resolution & unlimited penetration depth of MRI, realizing complementary dual‐modal precision tumor imaging, which effectively solves the limitations of single‐modal imaging in anatomical positioning and molecular level detection.

Wang et al.'s NGd‐AAs probe realizes dual amplification of fluorescence and MRI imaging. The aggregation‐induced fluorescence quantum yield is increased six‐fold, and the longitudinal relaxivity is increased 17‐fold, achieving high‐sensitivity dual‐modal imaging of tumors [132]. Xiao's OEGMA‐HPMA copolymer micelles have long‐circulation characteristics, realizing sustained tumor enrichment and stable dual‐modal imaging monitoring within 24 h after injection [133].

Meng et al.'s TB/SPIO@PS‐PEG nanoparticles (TSP NPs) integrate 655 nm AIE fluorescence and superparamagnetic iron oxide T_2_‐weighted MRI imaging function. The superparamagnetic properties produce tumor‐specific signal voids on T_2_‐weighted MRI, while red fluorescence provides intraoperative visual guidance. Longitudinal monitoring for 24 days shows sustained tumor retention, realizing quantitative evaluation of tumor therapeutic response through dual‐modal signal changes [137].

Targeted imaging further improves tumor diagnosis accuracy. T7‐AIE‐Gd nanoparticles significantly enhance the cellular uptake of hepatocellular carcinoma cells, realizing the visualization of subcentimeter tiny tumors that cannot be detected by conventional MRI [134]. BK@AIE NPs break through the blood–brain barrier, achieving precise imaging and positioning of intracranial glioblastoma, which provides a powerful tool for the diagnosis of refractory brain tumors [134].

In addition, Hua et al.'s Au_4_‐IO NP‐cRGD system realizes multimodal integrated diagnosis by combining fluorescence imaging, ECL detection, and radiotherapy imaging monitoring, further expanding the imaging application scope of magnetic responsive AIE materials [138].

##### 3.5.2.3 Electric Field‐Triggered AIE Ultra‐Precision Tumor Imaging

Electrical stimulation has ultra‐high temporal and spatial regulation precision, which can significantly enhance the fluorescence imaging signal of AIE materials, realize ultra‐micro tumor lesion imaging and early biomarker detection, and has unique advantages in precise tumor resection and early diagnosis.

Li et al.'s NIR‐II electroresponsive AIEgen (2TT‐m, oC6B) achieves ultra‐precise tumor imaging under electrical stimulation. Preoperative electrical stimulation (5 V/cm, 10 min) enhances fluorescence intensity 2.8‐fold, realizing the visualization of submillimeter tumor satellites (<0.5 mm) that are invisible under white‐light surgery. In orthotopic breast cancer models, the tumor‐to‐background ratio is increased from 4.2:1 to 11.8:1, significantly reducing postoperative local recurrence rate, which provides a new strategy for precise resection of borderline‐resectable tumors [135].

In terms of early tumor diagnosis, the AIE‐COF/CuS@Ag ECL sensor realizes ultra‐sensitive detection of glioma‐associated miRNA‐124‐3p with a detection sensitivity as low as 0.49 fM, which is three orders of magnitude higher than that of conventional fluorescence assays. Benefiting from plasmonic signal amplification effect, the sensor achieves 100% clinical sensitivity and 94.7% specificity, realizing early diagnosis of glioma through cerebrospinal fluid detection [133].

Moreover, the electric field‐triggered drug release AIE system can realize real‐time fluorescence imaging monitoring of drug delivery and release kinetics, forming a closed‐loop imaging guidance system for tumor treatment [135].

#### 3.5.3 Tumor Therapeutic Applications of Externally Triggered AIE Materials

Based on physical field‐responsive material design and precise imaging guidance, acoustic, magnetic, and electrical triggered AIE materials realize multimechanism synergistic tumor treatment, including SDT, magnetic hyperthermia/chemodynamic therapy, electric‐controlled drug delivery, and combined immunotherapy, achieving efficient inhibition of primary tumors and distant metastases.

##### 3.5.3.1 Ultrasound‐Triggered AIE Sonodynamic Therapy and Immunotherapy

SDT based on ultrasound‐responsive AIE materials is an emerging noninvasive tumor treatment modality, which uses ultrasound‐activated AIE sonosensitizers to generate ROS for deep tumor cell ablation. Ultrasound mechanical waves have ultra‐deep tissue penetration (>10 cm), which can effectively solve the depth limitation of traditional photodynamic therapy for deep‐seated tumors.

AIE sonosensitizers with optimized molecular structure achieve efficient ROS generation under ultrasound activation. The TCSVP sonosensitizer achieves a high ROS quantum yield of 0.89 under 1.0 MHz ultrasound irradiation, and the cationic structure targets tumor cell mitochondria efficiently [128]. The MeOTTMN AIE system combines ultrasound‐assisted optical activation to generate oxygen‐independent Type I ROS, realizing 87% inhibition of 4T1 breast tumors with 12 mm deep tissue treatment capability [139].

The most transformative advantage of AIE‐SDT is the induction of tumor ICD, realizing local treatment to systemic anti‐tumor immune response activation. The AIE/Biotin‐M system achieves 68.3% tumor cell apoptosis rate under clinical ultrasound parameters, with a ^1^O_2_ quantum yield of 0.43. The treated tumor cells show typical ICD characteristics: significantly up‐regulated CRT surface exposure, HMGB1 release, and ATP secretion, which effectively activate dendritic cell maturation and anti‐tumor T cell immune response [129].

Tian's TPA‐Tpy SDT system significantly increases tumor‐infiltrating CD8^+^ cytotoxic T cells and reduces immunosuppressive Tregs, realizing 83.5% dendritic cell maturation rate and 89% lung metastasis inhibition, showing excellent abscopal antitumor effect [125].

**FIGURE 16:**
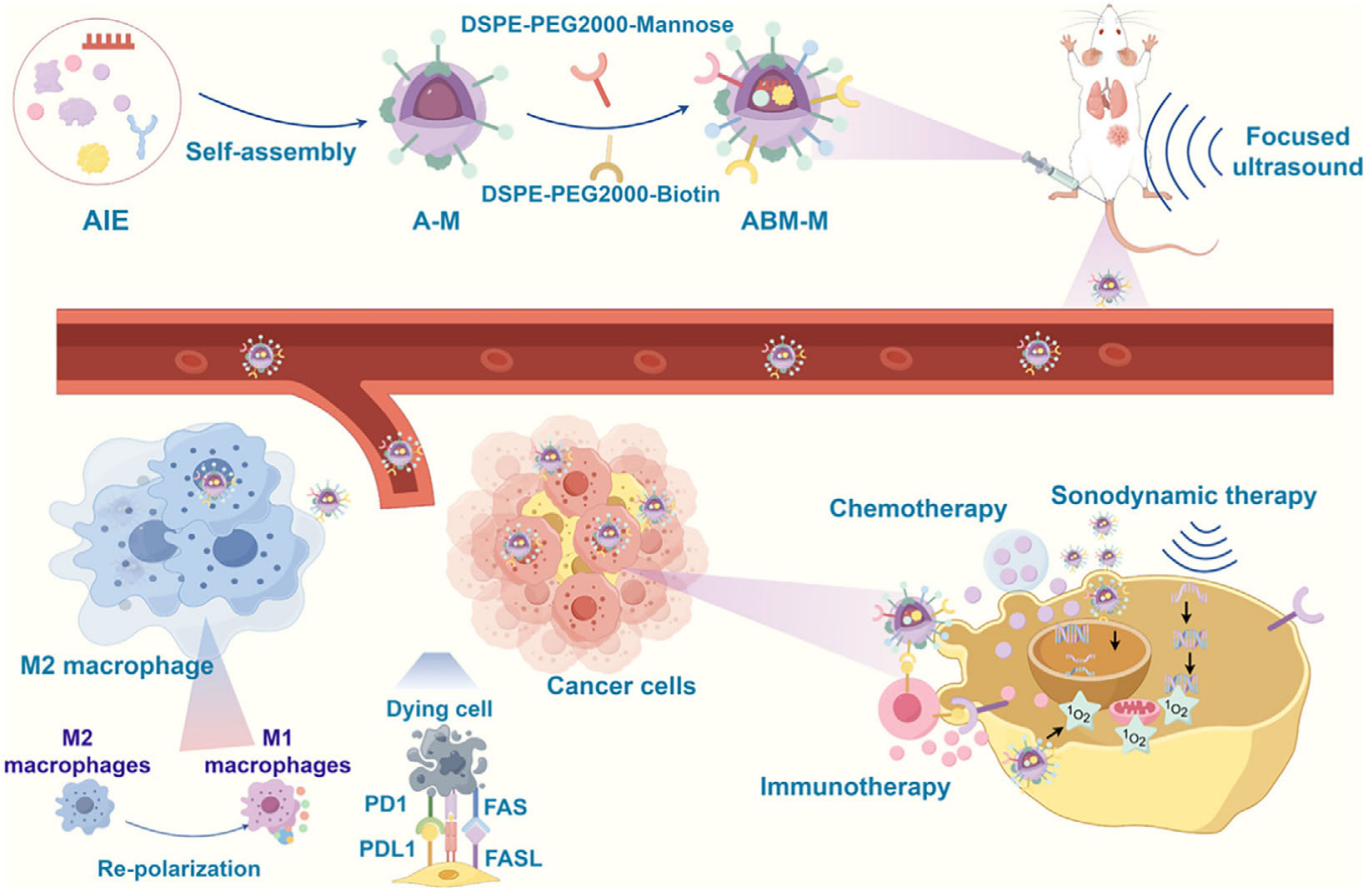
Immunogenic sonodynamic therapy for tumor cell growth inhibition and macrophage reprogramming. ABM‐M micelles target both tumor cells (biotin) and TAMs (mannose), inducing ICD and M2 → M1 repolarization. Combined with anti‐PD‐L1, this strategy achieves > 90% tumor inhibition through synergistic local and systemic effects. Reproduced with permission [140]. Copyright 2025, Acta Materialia Inc. Published by Elsevier Inc.

SDT combined with immune checkpoint blockade achieves synergistic therapeutic effect. Peng's ABM‐M dual‐targeting micelles reverse the polarization of tumor‐associated macrophages, convert pro‐tumor M2 macrophages to antitumor M1 phenotype, up‐regulate pro‐inflammatory cytokines and inhibit immunosuppressive mediators. Combined with anti‐PD‐L1 antibody, the tumor inhibition rate of CT26 colorectal cancer reaches 92.3%, which is significantly higher than that of single SDT treatment (Figure 16) [140].

##### 3.5.3.2 Magnetic Field‐Triggered AIE Synergistic Tumor Therapy

Magnetic responsive AIE materials realize multimodal synergistic tumor therapy including gene therapy, photothermal therapy, chemodynamic therapy, and immunotherapy under magnetic field guidance, with the advantages of precise tumor targeting and low systemic toxicity.

In terms of gene therapy, Hou et al.'s T7‐AIE‐Gd/siRNA nanocomplex achieves efficient targeted gene delivery. The T7 peptide significantly enhances the uptake of hepatocellular carcinoma cells, and the cationic AIE component protects siRNA from RNase degradation. The system achieves 68.3% Survivin gene silencing, down‐regulates antiapoptotic proteins, and improves tumor chemotherapy sensitivity by 4.2‐fold [138].

In terms of photothermal combined immunotherapy, Zhang's BK@AIE NPs integrate blood‐brain barrier penetration targeting and NIR photothermal therapy function. Under laser irradiation, the system induces a temperature increase of 28.5 °C in glioblastoma, triggers tumor ICD effect, activates CD8^+^ T cell immune response, and reprograms tumor‐associated macrophages to M1 antitumor phenotype. This local treatment activates systemic immune cascade, achieving 91.4% primary tumor inhibition and 78% distant tumor regression with obvious abscopal effect [132].

In terms of chemodynamic combined radiotherapy, Hua et al.'s Au_4_‐IO NP‐cRGD system responds to tumor acidic microenvironment to release Fe^2+^, catalyze Fenton reaction to generate cytotoxic hydroxyl radicals, and Au_4_ nanoclusters enhance radiotherapy sensitivity. The combined treatment of chemodynamic therapy and radiotherapy achieves 81.1% inhibition of 4T1 triple‐negative breast tumors, which is 2.3 times higher than that of single radiotherapy [138].

##### 3.5.3.3 Electric Field‐Triggered AIE Controlled Tumor Therapy

Electric field stimulation realizes spatiotemporally controllable tumor treatment through on‐demand drug release and precise therapeutic activation, solving the problems of uncontrolled drug release and low tumor targeting efficiency of traditional chemotherapy.

Wang et al.'s electric‐responsive TPE‐based polymer dots realize pulse‐controlled paclitaxel release. Under intermittent electric field stimulation, the drug release rate reaches 65%, which is significantly higher than 18% of passive diffusion. The electric‐controlled on‐demand dosing matches the peak tumor accumulation time of nanoparticles, achieving 89% 4T1 tumor growth rate, which is much higher than that of passive drug delivery formulations [135].

In addition, the electro‐enhanced AIE imaging‐guided precise surgery technology effectively reduces tumor recurrence. By improving the tumor‐to‐background imaging contrast, it realizes complete resection of tiny metastatic lesions, reducing the local recurrence rate of breast cancer from 38% to 7%, providing a new clinical transformation direction for precise tumor treatment [135].

Ma et al.'s multifunctional TDTMSB AIE agent realizes PDT‐PTT synergistic therapy under NIR‐II imaging guidance, with high Type I/II ROS generation efficiency and 40.5% photothermal conversion efficiency, achieving 94% efficient inhibition of 4T1 tumors, which verifies the excellent application potential of electric‐responsive AIE materials in synergistic tumor therapy [136].

#### 3.5.4 Key Challenges and Solution Strategies

##### 3.5.4.1 Core Existing Challenges

**FIGURE 17:**
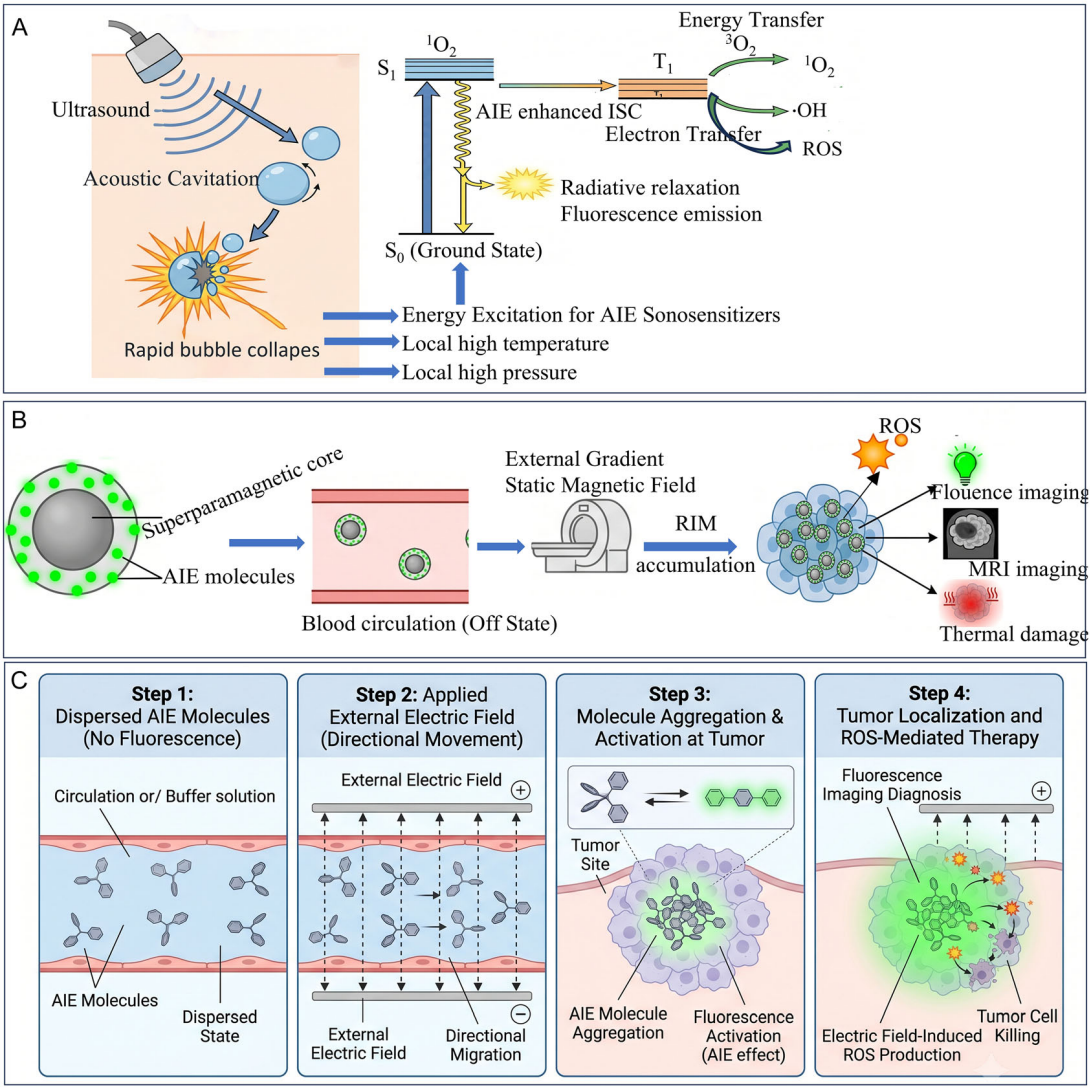
Schematic illustration of external stimuli‐responsive AIE materials for tumor theranostics. (A) Mechanism of ultrasound‐activated AIE sonosensitizers. Ultrasound triggers acoustic cavitation and bubble collapse, generating local high temperature/pressure to excite AIE molecules. Enhanced ISC promotes ROS production for sonodynamic therapy. (B) Magnetic targeting of superparamagnetic AIE nanoparticles. Under external magnetic field guidance, nanoparticles accumulate at the tumor site, where RIM turns on AIE fluorescence for multimodal imaging (fluorescence/MRI) and ROS‐mediated therapy. (C) Electric field‐driven tumor targeting and activation of AIE molecules. The applied electric field induces directional migration of dispersed AIE molecules to the tumor site, triggering aggregation and AIE activation for fluorescence imaging and electric field‐enhanced ROS therapy.

According to different external activation modes, externally triggered AIE tumor theranostic materials can be classified into ultrasound, magnetic field, and electric field‐responsive types. Figure 17 intuitively illustrates the design principles and performance advantages of external stimuli‐responsive AIE materials. The corresponding design strategies, response mechanisms, and representative platforms are summarized in Table 6. Despite the diversified advances of these physical field‐activated AIE systems, their clinical translation still faces systematic challenges in mechanism clarification, therapeutic performance, biosafety, and industrialization, which urgently requires targeted optimization and future technical innovation.

**TABLE 6:** Summary of externally triggered AIE materials for tumor theranostics.

External stimulus	Design principle	Response mechanism	Representative systems	References
Ultrasound stimulation	Optimize D–A and cationic AIE structures for high ROS production; construct targeted supramolecular assemblies and biomimetic nanoplatforms for tumor targeting and penetration	Ultrasound cavitation enhances ISC and triplet generation to produce Type I/II ROS for SDT; mechanical force cleaves sensitive bonds to trigger AIE aggregation, fluorescence activation and gas release	TPA‐Tpy TCSVP MeOTTMNAIEBiotin ABM‐M PAHN system TPA‐DPPy	[140, 127, 128, 129, 130, 131]
Magnetic field stimulation	Integrate AIE fluorophores with magnetic functional units and biocompatible matrices; introduce targeting/transcytosis structures for dual‐modal tumor imaging and accumulation	AIE aggregation restricts intramolecular rotation to activate fluorescence; clustered magnetic components improve MRI relaxation performance. Magnetic guidance promotes tumor enrichment and barrier penetration for synergistic theranostics	NGd‐Aas OEGMA‐HPMA T7‐AIE‐Gd BK@AIE NPs	[132, 133, 134, 137]
Electric field stimulation	Fabricate electric‐responsive D–A AIE molecules and conjugated polymers, and integrate electrochemical units for electrically regulated diagnosis and therapy	Electric field modulates molecular charge transfer and conformation to regulate fluorescence; electric pulses induce structural changes for controllable drug release and high‐sensitivity ECL detection	2TT‐m oC6B TDTMSBAIE	[133, 135, 136]

First, the molecular mechanism of physical field‐optical coupling is not fully clarified. The microcosmic regulation mechanism of ultrasound cavitation, magnetic field induction, and electric field stimulation on AIEgen molecular conformation, ISC and ROS generation needs further in‐depth exploration, which limits the targeted optimization of material performance.

Second, the penetration depth of partial physical stimuli is limited. The effective action depth of electrical and mechanical stimulation is less than 3 cm, which is difficult to meet the treatment needs of deep visceral tumors, restricting the application scope of electro‐responsive and mechano‐responsive AIE systems.

Third, the biosafety of long‐term and repeated physical stimulation is unclear. The long‐term neuromuscular and tissue damage effects of repeated electrical stimulation, as well as the cumulative biological toxicity of long‐term ultrasound/magnetic field combined with nanomaterials, lack systematic safety evaluation data.

Fourth, clinical translation barriers are prominent. Externally triggered AIE tumor therapy belongs to the combination of drug and medical devices, which involves complex regulatory approval pathways. In addition, the lack of standardized GMP‐compliant scalable synthesis technology restricts industrial production and clinical popularization.

Fifth, the single stimulus response mode has limited therapeutic synergy. Most current studies rely on single physical field activation, and the synergistic advantage of multiphysical field combined stimulation has not been fully exploited, resulting in insufficient treatment efficiency for refractory and metastatic tumors.

##### 3.5.4.2 Future Solution Strategies and Research Directions

Aiming at the above bottlenecks, future research on externally triggered AIE tumor materials should focus on mechanism innovation, technical upgrading, safety optimization, and clinical transformation, and the key development directions are summarized as follows:

First, develop AI‐assisted intelligent molecular design. Construct multistimuli‐responsive AIEgens with Boolean logic gate (AND/OR) response characteristics, realize precise selective activation of multiple physical field signals, avoid off‐target activation, and further improve tumor theranostic specificity.

Second, develop bioelectronic integrated implantable devices. Combine responsive AIE materials with miniature implantable electronic devices, realize long‐term, in situ and real‐time monitoring of recurrent tumors, and achieve early warning and intervention of tumor recurrence.

Third, innovate mechanobiology synergistic therapy strategies. Combine mechanical AIE activation technology with YAP/TAZ tumor mechanosignal pathway inhibitors, target the abnormal mechanical microenvironment of tumors, break tumor mechanical resistance, and improve the efficacy of physical therapy.

Fourth, establish standardized clinical transformation systems. Develop GMP‐compliant large‐scale synthesis and purification processes for AIE nanomaterials, formulate unified safety evaluation and efficacy detection standards for physical field combined therapy, and simplify the regulatory approval process for device‐drug combination products.

Fifth, explore multiphysical field synergistic activation modes. Integrate ultrasound deep penetration therapy, magnetic field targeted guidance and electric field precise regulation, develop orthogonal multistimuli responsive AIE systems, realize complementary advantages of different physical modalities, and improve the treatment effect of deep and metastatic tumors.

### 3.6 Multiple Synergistic Responsive AIE Materials for Tumor Diagnosis and Treatment

The single‐stimulus‐responsive AIE materials introduced in the previous chapters exhibit favorable application potential and research value in precise tumor diagnosis and treatment due to their simple response mechanisms and controllable preparation processes. However, such materials rely only on a single TME signal to activate their functions. Faced with the complex pathological characteristics of tumors, including multiparameter coupling, dynamic changes, and high heterogeneity, they present certain application limitations. Vulnerable to interference from inflammatory reactions and physiological metabolic fluctuations, these materials generally suffer from insufficient imaging specificity, off‐target activation, monotonous therapeutic modes, and limited therapeutic efficacy for deep lesions [43, 71, 100, 123]. To further compensate for the performance shortcomings of single‐stimulus‐responsive materials and expand the clinical application scenarios of AIE materials, researchers have constructed multistimulus synergistic responsive AIE systems by integrating two or more independent internal and external stimulus‐responsive units. These systems significantly improve the accuracy and environmental adaptability of tumor diagnosis and treatment through multisignal cross‐verification and multimechanism synergistic enhancement, emerging as a vital research direction in the field of precise tumor diagnosis and treatment.

#### 3.6.1 Design Principles and Classification

Multistimulus‐responsive AIE materials are generally designed based on the Boolean logic gate concept. By coupling various microenvironment‐sensitive functional groups with AIE luminescent skeletons, multicondition synergistic activation systems are constructed, in which fluorescence turn‐on, structural dissociation, and therapeutic function release are triggered only when multiple tumor characteristic signals are simultaneously matched [44, 125]. This multicheckpoint activation mechanism fundamentally avoids nonspecific responses caused by single abnormal signals and addresses the poor anti‐interference capability of single‐stimulus‐responsive materials. The integration of diverse independent responsive units enables the materials to adapt to multidimensional TME changes, achieving graded and precise functional activation in complex in vivo delivery and tumor intervention processes.

##### 3.6.1.1 Classification and Characteristics of Multistimulus Responsive Systems

Multistimulus‐responsive AIE systems integrate two or more independent responsive units and realize cross‐verification and hierarchical activation of tumor signals through cascade triggering mechanisms. Common response modes include pH/enzyme, hypoxia/temperature, pH/ultrasound, and other coupled combinations, which can be further matched with multiple intracellular microenvironmental signals (pH, GSH, enzymes, and hypoxia) and exogenous physical stimuli (magnetic field, ultrasound) to construct multilevel synergistic platforms. Among diverse designs, the pH/enzyme coupled system is the most mature and widely applied. Relying on the hierarchical response logic of “acid preactivation + enzyme‐mediated final activation,” the weakly acidic TME induces protonation and loosening of nanocarriers to expose hidden enzyme cleavage sites, and tumor‐specific highly expressed enzymes further complete structural activation and functional output, effectively preventing false activation in normal tissues and inflammatory regions [125]. Internal‐external coupled systems combine inherent tumor pathological signals with exogenous controllable physical stimuli, further improving the response stability of materials in dynamically changing complex microenvironments [43, 78]. With the increase of integrated responsive units, the system can achieve stricter multilayer logical screening, greatly reduce nonspecific loss and off‐target risks, and better adapt to the complex diagnosis and treatment scenarios of highly heterogeneous, refractory, and metastatic tumors [72, 77].

#### 3.6.2 Tumor Imaging Performance of Multistimulus‐Responsive AIE Materials

Compared with single‐stimulus‐responsive probes, multistimulus‐responsive AIE imaging materials rely on a multigate synergistic activation mechanism. Fluorescence is turned on only when multiple tumor characteristic signals coexist, which effectively reduces background noise, improves imaging signal‐to‐noise ratio and specificity, and overcomes the problems of high false‐positive rates and blurred lesion boundaries of single‐responsive probes. Such multisignal screening capability enables these materials to adapt to heterogeneous TMEs and achieve precise imaging of tiny lesions and deep tumors.

##### 3.6.2.1 Imaging Performance and Typical Applications of Multistimulus Responsive Systems

Among various dual‐responsive imaging systems, the pH/enzyme synergistic response mode exhibits the strongest adaptability and widest application range, serving as the most mature dual‐modal imaging strategy at present. Li et al. designed an MMP‐9/pH dual‐responsive AIE nanoprobe by modifying pH‐sensitive amino groups and MMP‐9 cleavable polypeptides on the surface of TPE‐based AIE skeletons. The probe remains in an ACQ state in normal neutral physiological environments. After accumulating liver cancer lesions, the weakly acidic TME induces protonation and stretching of nanoparticles to expose polypeptide cleavage sites. Highly expressed MMP‐9 further cleaves the skeleton structure to eliminate the aggregation quenching effect of AIE molecules, enabling specific near‐infrared fluorescence turn‐on. This dual‐lock activation mode effectively distinguishes inflammatory acidic environments from real TMEs, significantly increases the tumor‐to‐normal tissue signal ratio, and can accurately outline liver cancer infiltration boundaries for fluorescence‐guided intraoperative imaging [125].

To address the insufficient specificity of single hypoxia imaging, hypoxia/temperature dual‐responsive imaging systems realize tumor recognition based on dual metabolic abnormalities of tumors. Koide's team developed nitroimidazole‐modified thermosensitive AIE nanoprobes with both hypoxia reduction response and temperature sensitivity. Significant fluorescence enhancement occurs only under the coexistence of tumor hypoxia and local hyperthermia. The tumor hypoxic microenvironment triggers the reduction and activation of nitroimidazole groups, while local tumor hyperthermia optimizes the energy level transition efficiency of AIE molecules. The synergy of dual conditions achieves precise imaging of tumor metabolic abnormal regions. This probe effectively eliminates interference from physiological hypoxia and temperature fluctuations in normal tissues, exhibiting significantly higher recognition accuracy for deep hypoxic solid tumors than single hypoxia‐responsive probes [101].

Diversified multistimulus synergistic designs endow AIE probes with high‐precision imaging capability for complex tumors. Among them, the pH/enzyme synergistic response mode exhibits the strongest adaptability and widest application range, serving as the most mature dual‐modal imaging strategy at present. Li et al. designed an MMP‐9/pH dual‐responsive AIE nanoprobe by modifying pH‐sensitive amino groups and MMP‐9 cleavable polypeptides on the surface of TPE‐based AIE skeletons. The probe remains in an ACQ state in normal neutral physiological environments. After accumulating liver cancer lesions, the weakly acidic TME induces protonation and stretching of nanoparticles to expose polypeptide cleavage sites. Highly expressed MMP‐9 further cleaves the skeleton structure to eliminate the aggregation quenching effect of AIE molecules, enabling specific near‐infrared fluorescence turn‐on. This multilock activation mode effectively distinguishes inflammatory acidic environments from real TMEs, significantly increases the tumor‐to‐normal tissue signal ratio, and can accurately outline liver cancer infiltration boundaries for fluorescence‐guided intraoperative imaging [125]. To address the insufficient specificity of single‐index imaging, hypoxia/temperature synergistic systems realize tumor recognition based on dual metabolic abnormalities of tumors. Wang's team developed nitroimidazole‐modified thermosensitive AIE nanoprobes with both hypoxia reduction response and temperature sensitivity. Significant fluorescence enhancement occurs only under the coexistence of tumor hypoxia and local hyperthermia, which effectively eliminates interference from physiological hypoxia and temperature fluctuations in normal tissues [101]. For deep tumor imaging, pH/ultrasound internal‐external synergistic systems combine biochemical microenvironment recognition with exogenous physical regulation. Zhang et al. constructed gradient pKa‐regulated size‐shrinkable AIE nanoprobes that achieve adaptive particle size reduction in response to tumor gradient acidification. Assisted by ultrasound cavitation, the probes realize deep tumor penetration and fluorescence signal amplification, making up for the depth deficiency of traditional static biochemical imaging [78]. Further optimization of multiresponsive systems can achieve stricter signal screening. Typical pH/GSH/enzyme triple‐responsive AIE probes integrate acid‐sensitive groups, disulfide bonds, and enzyme‐cleavable fragments. The multilevel cascade activation of tumor weak acidity, high GSH concentration, and specific enzymes greatly reduces false‐positive probability, enabling accurate identification of tiny tumor lesions for early tumor screening [72]. In addition, magnetic field/ultrasound/pH multimodal synergistic systems integrate targeted enrichment, microenvironment activation and physical field enhancement. Fe_3_O_4_‐functionalized AIE nanoplatforms realize magnetic‐guided tumor accumulation, pH‐triggered fluorescence activation, and ultrasound‐assisted deep penetration optimization, achieving high‐precision multimodal imaging for primary and tiny metastatic lesions [77]. Novel multi‐index synergistic imaging systems such as quadruple‐responsive and logic‐encoded platforms have also been gradually developed, providing new technical support for tumor staged diagnosis and personalized imaging.

#### 3.6.3 Tumor Therapeutic Performance of Multistimulus‐Responsive AIE Materials

Single‐modal therapy based on single‐stimulus‐responsive AIE materials suffers from monotonous treatment mechanisms, obvious tumor drug resistance, and insufficient clearance of deep lesions. In contrast, multistimulus‐responsive AIE systems achieve synergistic enhancement of chemotherapy, photodynamic therapy, photothermal therapy, gas therapy, and immunotherapy through multisignal coupling. The multimechanism complementary mode can intervene tumor progression from multiple dimensions, effectively improving the overall therapeutic efficacy and biosafety of tumor treatment.

##### 3.6.3.1 Therapeutic Performance and Typical Applications of Multistimulus Responsive Systems

In dual‐responsive therapeutic systems, the pH/enzyme hierarchical response mode realizes temporally ordered precise drug release, serving as an effective strategy to reduce off‐target toxicity of traditional chemotherapy. Tian's team developed AIE drug‐loaded nanoplatforms with tandem acid‐sensitive bonds and MMP cleavable peptides. The carriers maintain stable structures without drug leakage in normal tissues. The weakly acidic TME loosens the carrier skeleton to expose enzyme cleavage sites, and tumor‐specific enzymes mediate complete carrier degradation to precisely release loaded chemotherapeutic drugs. Compared with ordinary nanomedicines, this hierarchical drug release mode achieves high local drug accumulation in tumors, reduces systemic toxic and side effects, and exhibits significant tumor inhibition effects on subcutaneous xenografts and orthotopic liver cancer with improved biological safety [125].

Aiming at the common treatment tolerance of hypoxic solid tumors, hypoxia/temperature dual‐responsive synergistic therapeutic systems break the limitations of single therapy through multimechanism complementation. Wu's group constructed AIE therapeutic systems integrating hypoxia‐activated photosensitizers and thermosensitive CO‐releasing units. The hypoxic tumor region activates photosensitizers to generate reactive oxygen species for basic photodynamic therapy. Mild tumor photothermal stimulation triggers CO gas release, inhibits HSP expression, and reverses tumor thermal tolerance, realizing synergistic therapy of low temperature photothermal therapy, photodynamic therapy, and gas therapy. This system effectively solves the problems of invalid photodynamic therapy in hypoxic tumors and normal tissue damage caused by high‐temperature photothermal therapy, significantly improving the eradication rate of hypoxic solid tumors [102].

Multistimulus‐responsive AIE therapeutic systems achieve multimechanism synergistic treatment through multisignal coupling, effectively overcoming the limitations of single‐modal therapy such as insufficient efficacy and tumor drug resistance. The pH/enzyme multiresponsive drug delivery design is a classic strategy to reduce chemotherapy off‐target toxicity. Zhao's team developed AIE nanoplatforms with tandem acid‐sensitive bonds and MMP cleavable peptides, which remain stable in normal tissues and achieve hierarchical degradation and precise drug release targeting TMEs, reducing systemic side effects and improving in vivo antitumor efficacy [125]. For hypoxic tumor treatment resistance, hypoxia/temperature synergistic systems integrate hypoxia‐activated photosensitizers and thermosensitive gas‐releasing units. The combination of photodynamic therapy, low‐temperature photothermal therapy and gas therapy reverses tumor thermal tolerance and compensates for the insufficient efficacy of single treatment modalities in hypoxic tumors [102].

To solve the problem of difficult deep tumor treatment, pH/ultrasound synergistic systems realize TME‐triggered structural remodeling, and ultrasound cavitation further promotes deep penetration and burst drug release, achieving combined mechanical and chemical treatment for deeply invasive tumors and reducing recurrence risk [78]. On the basis of dual‐index synergy, multiresponsive systems with more integrated functional units exhibit better performance in reversing tumor drug resistance and activating antitumor immunity. The pH/GSH/enzyme triple‐responsive system can hierarchically regulate tumor redox homeostasis, block cell cycle and reverse multidrug resistance, achieving efficient inhibition of drug‐resistant tumors [72]. Furthermore, magnetic field/ultrasound/pH multimodal platforms integrate targeted delivery, microenvironment‐responsive treatment and external field synergistic enhancement, which can ablate local tumors via photodynamic/photothermal therapy, induce ICD, and activate systemic anti‐tumor immune responses to inhibit tumor metastasis. The degradable structural design also ensures good biosafety of the nanoplatforms after treatment [77].

#### 3.6.4 Key Challenges and Future Perspectives

Compared with single‐stimulus‐responsive AIE materials, multistimulus‐synergistic‐responsive systems have three core advantages. First, the multigate activation mechanism significantly reduces nonspecific activation and off‐target risks, improving diagnosis and treatment specificity. Second, multimodal therapeutic synergy breaks the efficacy bottleneck of single therapy and overcomes tumor heterogeneity and drug resistance. Third, multidimensional structural design optimizes tumor accumulation, deep penetration, and in vivo metabolism simultaneously, balancing therapeutic efficacy and biological safety [125].

Currently, the field faces three core bottlenecks. The integration of multifunctional units leads to complex synthesis processes and poor batch repeatability. The multimodule synergistic structure–activity relationship and long‐term in vivo biosafety lack systematic quantitative research. The absence of unified industry evaluation standards hinders horizontal performance comparison and restricts clinical translation progress [77].

Future research can focus on three breakthrough directions. Artificial intelligence‐assisted molecular design can be adopted to optimize multimodule coupling structures, simplify synthesis processes, and improve batch stability. An integrated evaluation system combining organoids and multiscale animal models can be constructed to establish standardized performance and safety evaluation protocols. Modular preparation processes can be optimized to build GMP‐compliant production systems, accelerating the clinical translation and industrialization of multistimulus synergistic responsive AIE materials for tumor diagnosis and treatment [77, 115].

## 4 Critical Comparison and Translational Perspectives of Stimuli‐Responsive AIE Theragnostic Systems

Before systematically comparing different stimuli‐responsive AIE strategies, we summarize the core performance parameters, corresponding experimental conditions, adopted characterization methods, advantages and limitations of representative AIE materials reported in this review in Table 7. These quantitative data cover ROS yield, fluorescence quantum yield, imaging performance, antitumor efficacy, cellular uptake, and drug release behavior, which provide a solid data basis for the following discussion.

**TABLE 7:** Summary of key performance indicators, experimental conditions, characterization methods and properties of representative stimuli‐responsive AIE materials for tumor theranostics.

Indicator	Material	Experimental conditions	Characterization method	Advantages	Limitations	References
ROS yield	SC3, ∼5‐fold increase in ROS yield compared to control	Hypoxia, light irradiation	ROS fluorescent probe assay	Maintains enhanced ROS generation under hypoxic conditions	Increased ROS yield alone does not directly translate to therapeutic efficacy without in vivo validation	[14]
	AIE/Biotin‐M, ΦΔ = 0.72	Ultrasound activation, biotin targeting (SDT)	^1^O_2_ quantum yield measurement	Combines targeting and deep‐tissue SDT capability	Depends on biotin receptor expression and ultrasound distribution heterogeneity	[22]
	TPE‐Ph‐NH_2_, ΦΔ = 0.82 (5.5× vs. neutral pH)	pH 5.0, PDT irradiation	^1^O_2_ quantum yield measurement	Significantly enhanced ^1^O_2_ generation in acidic tumor microenvironment	Strong dependence on local pH and light accessibility limits effectiveness in heterogeneous tumors	[7]
Fluorescence quantum yield	CaCO_3_@BSA‐TPE‐Qu^+^, QY increased from 0.12 to 0.68; SBR = 9.7:1	Acid‐triggered CaCO_3_ dissolution and AIE activation	Fluorescence spectroscopy/QY measurement	High signal‐to‐background ratio via stimulus activation	Performance depends on sufficient local acidity; batch reproducibility of hybrid structure may be challenging	[27]
Fluorescence enhancement	P‐TN‐Dox@CM, ∼7.2‐fold fluorescence enhancement	pH/temperature dual‐responsive nanogel	Fluorescence intensity analysis	Enables real‐time monitoring of therapeutic process	Complex membrane‐coated nanostructure may hinder reproducibility and large‐scale fabrication	[25]
Imaging depth	PE‐Py‐PAI, ∼10.2 mm depth, 78 μm resolution	Photoacoustic/fluorescence dual‐modality imaging	PA/fluorescence imaging	Combines deep penetration with high spatial resolution	Strong dependence on dual‐modality instrumentation limits clinical translation	[35]
	TPE‐BODIPY, ∼12 mm penetration (NIR‐II)	NIR‐II imaging	NIR‐II fluorescence imaging	Reduced background and improved deep‐tissue imaging	Primarily imaging‐oriented; limited therapeutic functionality	[38]
Uptake/accumulation	Targeted AIE systems, 3–10× tumor accumulation	RGD/biotin/TPP targeting	In vivo fluorescence imaging/biodistribution	Enhanced active targeting and tumor accumulation	Strong dependence on receptor expression and tumor heterogeneity	[18]
Cellular uptake	CS‐BT‐HBS‐CB, ∼5.7× increased binding	Biotin receptor‐mediated uptake	Cellular uptake assay	Efficient receptor‐mediated internalization	Limited applicability in tumors with low biotin receptor expression	[29]
	NAB@DD‐DC, ∼6× increase; Zeta potential −25 → +15 mV	pH‐triggered charge reversal	Uptake assay/Zeta potential measurement	Enhanced cellular uptake via charge conversion	Positive charge exposure may increase nonspecific adsorption and toxicity	[26]
Drug release	P‐TN‐Dox@CM, ∼80% DOX release in 5 min	Acidic pH	In vitro release assay	Rapid drug release at tumor site	Burst release may cause premature leakage and short therapeutic window	[25]
	CE‐TPEHy‐NMs, 11%/50%/80% release at pH 7.4/6.5/4.5	pH‐dependent release	Release profile analysis	Clear pH‐selective release behavior	Single pH‐trigger mechanism may be insufficient under complex in vivo conditions	[42]

### 4.1 Critical Comparison of the Five Stimuli‐Responsive Strategies

From the fundamental perspective of AIE, its performance is not merely defined by “emission upon activation,” but rather by how the triggering process regulates the coupling between molecular aggregation states and restriction of RIM. Therefore, the key distinction among different stimulus‐responsive strategies lies not in whether a response can be achieved, but in whether the process can efficiently and controllably drive aggregation–disaggregation transitions or rigidification, thereby enabling high signal‐to‐noise ratios and amplified outputs.

pH‐responsive AIE systems typically rely on protonation/deprotonation to modulate molecular hydrophilicity/hydrophobicity or charge distribution, thereby inducing aggregation or disassembly to activate fluorescence. These systems are facile in design and widely applicable, making them among the earliest and most explored strategies. However, a fundamental limitation is that pH‐induced modulation is generally gradual rather than threshold‐like, making it difficult to achieve an ideal “off–on” switching behavior. This results in limited fluorescence contrast and relatively high background signals. Although AIE effectively overcomes ACQ, pH stimuli do not fully exploit the intrinsic advantage of AIE in achieving high‐gain switching. Thus, from the perspective of mechanism–performance matching, pH responsiveness represents a low‐barrier and easily implementable strategy, whose prevalence in the literature may be somewhat overestimated. It may be more suitably employed as an auxiliary modulation mechanism rather than a primary trigger.

Redox‐responsive AIE systems, in contrast, more closely align with the core strengths of AIE. These systems typically utilize GSH‐triggered bond cleavage (e.g., disulfide bonds) or ROS‐induced structural rearrangements to induce a transition from dispersed (weakly emissive) to aggregated (strongly emissive) states, or vice versa. The key advantage lies in the fact that redox reactions often occur at the level of chemical bond transformation, providing a discrete switching behavior and significantly enhanced on/off contrast. This makes them particularly suitable for constructing activatable probes. However, this advantage is accompanied by an inherent limitation: such processes are often irreversible, leading to reduced controllability and limiting their applicability in dynamic monitoring or repeated‐response systems. Consequently, redox‐responsive AIE systems are more suitable for one‐time activation scenarios, such as drug release or endpoint imaging, representing a strategy with strong signal output but relatively constrained application scope.

Enzyme‐responsive AIE systems represent the most refined and biologically specific class of designs. By incorporating enzyme‐cleavable moieties (e.g., peptide substrates) into AIEgens, highly selective “structural unlocking” can be achieved, which subsequently induces molecular rigidification or localized aggregation, thereby activating fluorescence. A key advantage is the catalytic amplification effect of enzymes, enabling significant signal generation even at low target concentrations. In addition, enzyme‐mediated activation often occurs locally, providing spatial precision that synergizes with the aggregation‐induced emission mechanism to yield low background and high signal amplification. As such, enzyme‐responsive systems exhibit strong potential in applications such as precise diagnostics, intraoperative imaging, and molecular classification. Nevertheless, their structural complexity introduces challenges in synthesis, in vivo stability, and potential immunogenicity, which remain underappreciated obstacles for clinical translation.

Thermoresponsive AIE systems operate by modulating the degree of intramolecular motion (i.e., RIM) through temperature changes, which is conceptually consistent with the AIE mechanism. However, physiological temperature variations are typically insufficient to induce substantial changes in RIM, and thus most systems rely on external heating sources, such as photothermal or magnetothermal effects. This effectively shifts their nature from endogenous to exogenously coupled responses. Moreover, thermal effects are generally nonspecific and global, making precise molecular‐level control difficult to achieve. Therefore, thermoresponsive AIE systems are more suitably employed as auxiliary functional modules, such as feedback elements in PTT, rather than as independent triggering mechanisms.

Exogenous stimulus‐responsive AIE systems (e.g., ultrasound, electric fields, and magnetic fields) can be evaluated based on whether they directly regulate aggregation behavior or RIM. Among these, ultrasound is the most representative, as it can induce nanostructural reorganization or molecular aggregation via cavitation and mechanical effects, thereby achieving a form of “physically driven” fluorescence activation that closely aligns with the AIE mechanism. In addition, its deep tissue penetration provides practical advantages for applications in deep‐seated tumors. In contrast, magnetic field‐based systems primarily rely on magnetic carriers for targeting or thermal effects, with only indirect influence on AIE processes, while electric field‐responsive systems are currently limited by challenges in in vivo control and insufficient response efficiency. As such, only ultrasound demonstrates strong mechanistic coupling with AIE, whereas magnetic and electric field strategies remain largely auxiliary or exploratory.

Overall, these stimulus‐responsive strategies exhibit a certain degree of hierarchical differentiation in AIE systems. Enzyme‐ and ultrasound‐responsive approaches show relatively strong alignment with the “aggregation modulation–signal amplification” paradigm and are generally considered to hold promising potential. Redox‐responsive systems offer high signal output but are constrained by their irreversibility. In contrast, pH‐ and electric field‐responsive strategies display certain limitations that warrant further systematic evaluation, while temperature and magnetic field approaches are more often employed as complementary regulatory tools. Taken together, the future development of AIE materials may rely less on expanding the diversity of stimuli, and more on selecting appropriate triggering mechanisms and achieving precise regulation of aggregation states and RIM within multiresponsive systems.

### 4.2 Translational Barriers and Prospects of Stimuli‐Responsive AIE Theranostic Materials

From a translational perspective bridging laboratory research and clinical practice, the five categories of stimuli‐responsive AIE theranostic platforms face multiple intertwined bottlenecks during their progression from bench to bedside. These challenges necessitate systematic evaluation across multiple dimensions, including biosafety, biodegradation, in vivo clearance, immunogenicity, off‐target activation, experimental reproducibility, as well as large‐scale manufacturing and regulatory compliance [141].

At the level of fundamental biosafety, most AIE skeletons and inorganic components may induce chronic or subacute organ toxicity due to long‐term retention in vivo, particularly through accumulation in the liver, spleen, and reticuloendothelial system, thereby limiting their suitability for repeated administration in long‐term disease monitoring. In addition, certain heavy‐atom‐doped AIE systems designed to enhance ROS generation may introduce potential carcinogenic risks and metabolic disturbances, further increasing uncertainty in clinical applications [142].

Regarding biodegradation and in vivo clearance, the metabolic fate of AIE nanomaterials is largely governed by particle size, surface charge, and hydrophilic modification. Smaller constructs tend to undergo rapid renal clearance, whereas larger particles are more likely to be captured by the liver and exhibit prolonged retention. However, most existing AIE systems still lack controllable degradation behaviors that match physiological excretion kinetics, resulting in insufficient elimination even after completing their diagnostic or therapeutic functions [143].

Unpredictable immunogenicity constitutes another critical barrier to translation. Exogenous AIE nanocomposites, along with their conjugated targeting peptides or biopolymer coatings, may activate the innate immune system, inducing macrophage polarization and pro‐inflammatory cytokine release. This process can trigger nonspecific “turn‐on” fluorescence in inflamed tissues, significantly increasing false‐positive imaging signals and compromising tumor‐targeting accuracy [144]. Such inflammation‐induced off‐target luminescence represents a common limitation across pH‐, enzyme‐, and redox‐responsive AIE systems, as inflammatory lesions often share similar microenvironmental features—such as acidity, elevated GSH levels, or enzyme overexpression—with tumor tissues, leading to erroneous probe activation outside tumor sites [145].

In terms of experimental reproducibility, a substantial gap exists between laboratory studies and preclinical or clinical settings. Most reported AIE performance data are obtained under highly standardized in vitro or small‐animal conditions, typically using homogeneous tumor xenograft models. In contrast, human primary tumors are highly heterogeneous, with significant spatial variations in biomarker levels (e.g., MMP or GSH) between tumor cores and invasive margins. This heterogeneity leads to considerable fluctuations in fluorescence activation efficiency and therapeutic outcomes when translated to clinical samples [146].

Moreover, nearly all current AIE materials remain at the milligram‐scale batch synthesis stage and are difficult to scale up into continuous, GMP‐compliant industrial production [147]. During scale‐up, key quality parameters such as particle size distribution, zeta potential, and drug loading efficiency often exhibit significant drift, resulting in poor batch‐to‐batch consistency and failure to meet the stringent quality control requirements of regulatory agencies such as the FDA and EMA [148]. From a regulatory standpoint, AIE‐integrated theranostic systems are classified as hybrid products combining imaging contrast agents and therapeutic nanomedicines, leading to ambiguous categorization within existing drug and medical device frameworks. The lack of standardized evaluation criteria for in vivo toxicology, pharmacokinetics, and long‐term safety further prolongs approval timelines and hinders clinical translation [4].

Rational carrier modification is another practical route to alleviate the above‐mentioned translational obstacles. Cell membrane coating helps prolong blood circulation and tumor accumulation while mediating immune regulation. A range of biocompatible carriers including calcium phosphate microspheres, PLGA and shellac‐modified carbon nanotubes are available for encapsulating enzyme‐sensitive AIE luminogens [149]. Calcium phosphate can immobilize nucleic acids on its surface to inspire structural optimization of delivery vehicles [150]; adjustable fabrication parameters of PLGA enable rational particle size control and reduced nonspecific clearance in vivo [151]; carbon nanotubes noncovalently coated with shellac feature better dispersion and lower cytotoxicity, serving as ideal scaffolds for AIE loading [152].

To address these multifaceted challenges, multilock (dual‐ or multiresponsive) molecular design has emerged as a mainstream optimization strategy. By integrating two or more endogenous tumor biomarkers, AIE activation can be restricted to conditions where multiple pathological signals coexist, thereby significantly reducing nonspecific activation. Meanwhile, the incorporation of degradable aliphatic linkages and renal‐clearable structural engineering has been widely adopted to shorten in vivo retention and mitigate chronic toxicity risks, paving a feasible path toward the clinical translation of next‐generation AIE theranostic systems [153].

## Author Contributions

**Zhaohua Xu**: writing‐review and editing, writing – original draft. **Deyue Kong**: review draft. **Xiaoru Li**: writing – original draft. **Miao Zhu**: writing – original draft. **Haili Yan**: writing – review and editing, funding acquisition. **Chenyang Zhang**: writing – original draft, funding acquisition. **Xiaochun Wang**: writing – review and editing. **Duiping Feng**: funding acquisition. **Jiangfeng Du**: writing – review and editing, supervision, conceptualization, funding acquisition.

## Funding

This work was supported by the “Sanjin Talent” Leading Scientists and Innovators Program, National Natural Science Foundation of China (32201160, 52402348), Shanxi Applied Basic Research Project (202303021221223), Shanxi Provincial Clinical Research Center for Interventional Medicine (202204010501004), Central Government Guiding Local Science and Technology Development Funds (YDZJSX20231B010).

## Conflicts of Interest

The authors declare no conflicts of interest.

## Data Availability

Data sharing not applicable to this article as no datasets were generated or analysed during the current study.
